# Extracellular matrix remodeling and immune reprogramming drive residual tumor progression of liver cancer after incomplete microwave ablation

**DOI:** 10.1002/1878-0261.70337

**Published:** 2026-09-22

**Authors:** Yu Liu, Yubei He, Nikolaus Berndt, Michael Mülleder, Kathrin Textoris‐Taube, Jing Guo, Matthias Stechele, Justus Ramtke, Shraga Nahum Goldberg, Lynn Jeanette Savic

**Affiliations:** ^1^ Department of Radiology Campus Virchow‐Klinikum, Charité – University Medical Center Berlin, Corporate Member of Freie Universität Berlin and Humboldt‐Universität zu Berlin Berlin Germany; ^2^ Experimental Clinical Research Center (ECRC) at Charité – University Medical Center Berlin and Max‐Delbrück‐Centrum für Molekulare Medizin (MDC) Berlin Germany; ^3^ Department of Molecular Toxicology German Institute of Human Nutrition Potsdam‐Rehbruecke (DIfE) Nuthetal Germany; ^4^ Department of Radiology Charité – University Medical Center Berlin, Corporate Member of Freie Universität Berlin and Humboldt‐Universität zu Berlin Berlin Germany; ^5^ Core Facility High‐Throughput Mass Spectrometry, Charité – University Medical Center Berlin, Corporate Member of Freie Universität Berlin and Humboldt‐Universität zu Berlin Berlin Germany; ^6^ Department of Radiology University Hospital, LMU Munich Munich Germany; ^7^ Goldyne Savad Institute of Gene Therapy and Division of Image‐Guided Therapy and Interventional Oncology, Department of Radiology Hadassah Hebrew University Medical Center Jerusalem Israel; ^8^ Berlin Institute of Health at Charité – University Medical Center Berlin Berlin Germany; ^9^ Department of Radiology and Nuclear Medicine Otto‐von‐Guericke University Magdeburg Magdeburg Germany

**Keywords:** cellular signal pathways, immune cells, liver cancer, microwave ablation, proteomic analysis, residual tumor

## Abstract

Although microwave ablation (MWA) constitutes an effective targeted treatment for liver cancer, the mechanisms driving high recurrence rates remain poorly understood. To elucidate these processes, we established an incomplete MWA (iMWA) model in rabbit VX2 tumors and conducted proteomic profiling of residual tumors at Days 3 (D3) and 14 (D14) postablation. By integrating genomic profiles from mouse radiofrequency ablation (RFA) models, we identified 81 and 92 upregulated proteins at D3 and D14, respectively. Functional enrichment analysis (STRING and Enrichr) revealed acute injury response and extracellular matrix (ECM) remodeling predominating at D3, then markedly declining by D14. Conversely, pathways associated with signal transduction, tumorigenesis, and immune pathways were substantially upregulated at D14. Specifically, protein–protein interaction (PPI) analysis revealed that macrophage remodeling and immune checkpoint networks are functionally coupled at D14 to orchestrate an immunosuppressive microenvironment. Stouffer analysis confirmed the shift from matrix remodeling at an early stage to late‐stage oncogenic signal transduction and immune suppression at progression. Overall, these findings characterize the dynamic mechanisms of postablation residual tumor growth and highlight potential therapeutic targets to inhibit protumorigenic pathways while enhancing antitumor immunity.

## Graphical abstract

Incomplete microwave ablation (iMWA) of liver cancer triggers a biphasic progression in residual tumors. At Day 3, the microenvironment is characterized by acute inflammatory responses and extracellular matrix (ECM) remodeling. By Day 14, a profound shift occurs toward oncogenic signal transduction and immunosuppression, marked by macrophage polarization and immune checkpoint activation, ultimately driving residual tumor progression.

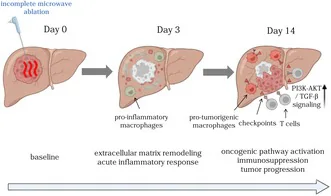

**Abbreviations**
CT: computed tomography
D0: baseline (without treatment)
D1: Day 1
D3: Day 3
D6: Day 6
D7: Day 7
DDA: data‐dependent acquisition
ECM: extracellular matrix
FDR: false discovery rate
GO: gene ontology
HCC: hepatocellular carcinoma
HCD: higher‐energy collisional dissociation
H&E: hematoxylin and eosin
iMWA: incomplete microwave ablation
LC: liquid chromatography
log2FC: Log2 fold changes
MCL: Markov Cluster Algorithm
MRE: magnetic resonance elastography
MRI: magnetic resonance imaging
MWA: microwave ablation
OR: odds ratio
PPI: protein–protein interaction
RFA: radiofrequency ablation
scRNAseq: single‐cell RNA sequencing
VEGF: vascular endothelial growth factor

## 1 Introduction

Primary liver cancer ranks as the sixth most common tumor worldwide and the fourth leading cause of tumor‐related mortality [1]. A variety of treatment modalities shape the current therapeutic arsenal, including surgical resection, liver transplantation, locoregional treatments including thermal and other forms of ablation, targeted and immunotherapy [2]. Thermal ablation has proven safe and equally efficacious to surgery for early‐stage hepatocellular carcinoma (HCC) and liver metastases [3, 4]. Microwave ablation (MWA), a widespread heat‐based ablation technique, has been applied since the 1990s. Using imaging guidance such as computed tomography (CT) or ultrasound to localize the lesion, a needle‐like antenna is positioned within the tumor tissue to emit electromagnetic waves at 915 MHz or 2.45 GHz [5]. These waves cause collisions with ions and water molecules near the antenna, generating frictional heat that rapidly raises the surrounding temperature. The resultant high temperatures induce coagulative necrosis of the tumor tissue and surrounding areas in the ablation zone [6]. In clinical practice, clinicians aim to completely cover the tumor and a safety peri‐ablational margin of at least 5 mm surrounding the tumor [7]. However, local residual tumor growth remains a significant concern and has been associated with larger tumor size, inadequate ablation margins, and proximity to major vessels, where heat is dissipated by flowing blood (so‐called heat‐sink effect) [8]. Compared to its predecessor, radiofrequency ablation (RFA), MWA can more rapidly achieve larger ablation zones per insertion and thus it is frequently used for larger tumors [9]. Overall, recurrence rates of approximately 20% for lesions < 5 cm and nearly 40% for larger ones are encountered for MWA [10].

The precise mechanisms driving and relative causes of early residual tumor growth following MWA and other interventional oncologic procedures have been debated and studied for decades but remain poorly understood. Existing hypotheses include that thermal ablation may elevate intratumoral pressure, facilitating the shedding and dissemination of viable tumor cells, which can subsequently seed and proliferate in adjacent areas [11]. Furthermore, MWA is thought to provoke a sustained inflammatory milieu, characterized by the persistent release of vascular endothelial growth factor (VEGF), and other protumorigenic factors, thereby fostering a permissive environment for tumor progression [12].

Evolving analytic techniques including genomic and proteomic analyses may help better understand the signaling pathways and cellular responses triggered by MWA that are involved in residual tumor growth. Yet, due to ethical considerations and complication risks of biopsies, human studies investigating these mechanisms are usually limited. In addition, according to the BCLC (Barcelona Clinic Liver Cancer) guidelines [13], a biopsy is not mandatory for HCC diagnosis when typical imaging hallmarks are identified on multiphasic CT or MRI in high‐risk patients. Thus, clinical research is often restricted to peripheral blood samples or tissue samples obtained before and during ablation, which do not represent the optimal timeframe to assess the direct effects where they are greatest [14]. Animal models can potentially overcome these limitations, enabling *in situ* detailed analysis of the signaling pathways and immune responses during postablation residual tumor growth, thus providing critical insights for developing targeted interventions.

This study aimed to elucidate the molecular mechanisms driving residual tumor growth following incomplete microwave ablation of liver cancer using a rabbit liver tumor model. To address this, we employed the translational rabbit VX2 liver tumor model. While VX2 is technically an implantable squamous cell carcinoma rather than a primary liver cancer, it is a well‐established surrogate in interventional oncology. This model replicates key hallmarks of human HCC, such as hypervascularity and high glycolytic activity, which are essential for studying thermal ablation responses. Furthermore, this immunocompetent and larger scale animal platform provides unique translational advantages over murine models, as its anatomical dimensions accommodate standard clinical MWA and MRI equipment while allowing for high‐fidelity histopathological and proteomic mapping to clearly distinguish the viable residual margin from the central necrotic zone. We analyzed residual viable tumor collected at D3 and D14 postablation to capture the dynamic alterations in subacute and long‐term signaling pathways through comprehensive proteomic profiling. By integrating these data with transcriptomic findings from complementary animal models, this study sought to uncover the key molecular processes underlying postablation residual tumor growth and to identify potential molecular biomarkers and therapeutic targets to mitigate residual tumor growth and enhance treatment efficacy.

## 2 Materials and methods

### 2.1 Study design and rabbit liver tumor model of incomplete microwave ablation (iMWA)

**Fig. 1:**
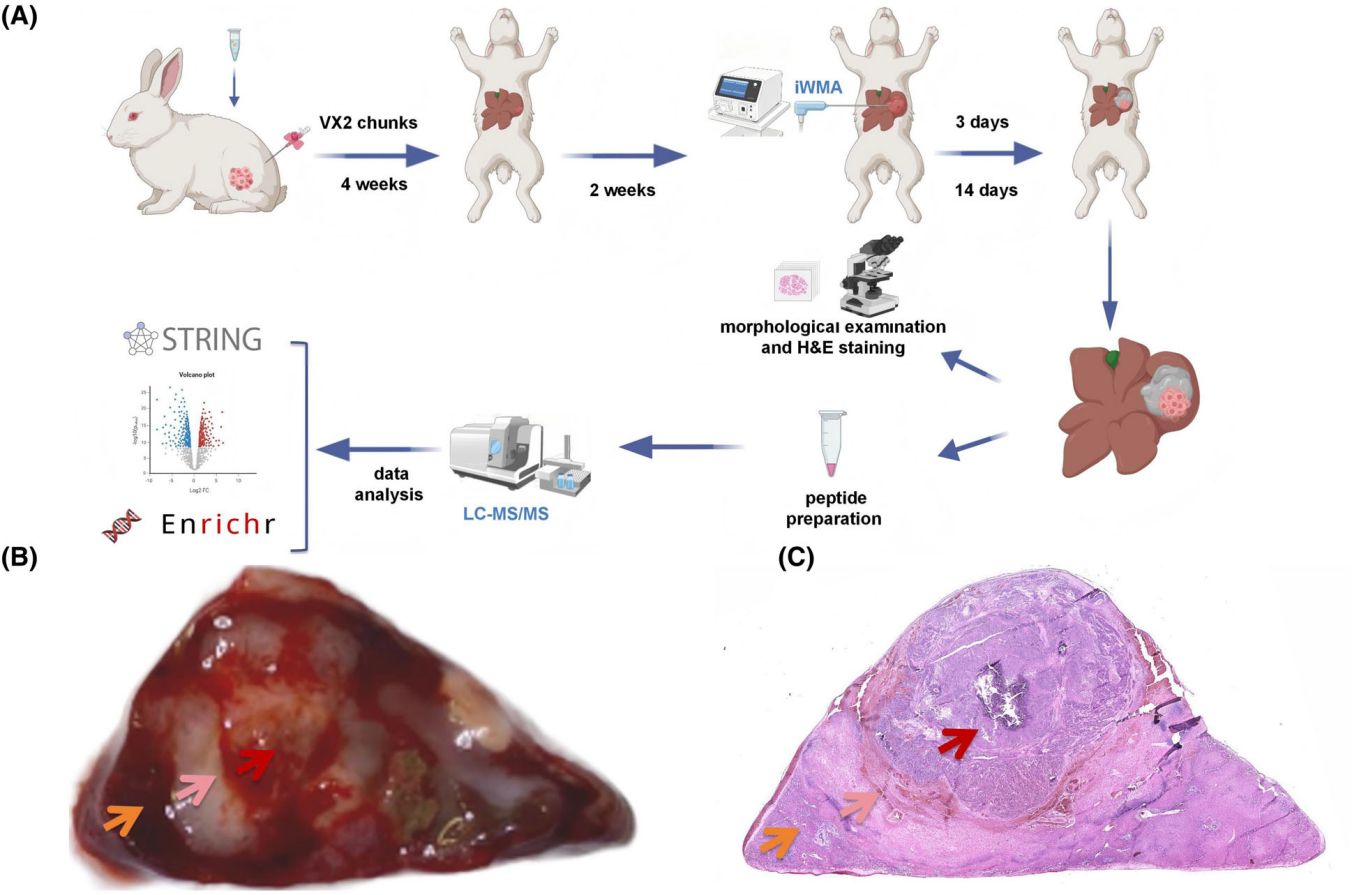
Project design overview and **t**issue characterization. The scheme of the experimental procedures (figure created with BioRender.com). Group 1/Control group at Day 0 (D0): received no treatment, serving as tumor baseline. Group 2/subacute group at Day 3 (D3): underwent incomplete microwave ablation (iMWA), targeting at least 50% of the tumor. Tissues were collected at 3 days postablation. Group 3/long‐term group at Day 14 (D14): underwent the same treatment, and the liver tissues were collected at 14 days postablation. LC–MS/MS: Liquid Chromatography–Tandem Mass Spectrometry (A). The freshly harvested liver (B) and Hematoxylin and Eosin (HE) staining (C). Red arrows indicate the center of ablation, the blue arrows indicate the viable residuals, and the green arrows indicate the adjacent liver tissues.

The overall study design and experimental workflow are illustrated in Fig. 1A. A summary of the primary methods and results is presented in the main text, while full methodological details, supporting datasets, and additional analyses are available in the supplemental material.

Animal Models: All animal experiments and related procedures were performed in accordance with the German Animal Welfare Act (Tierschutzgesetz) and relevant national legislation. The study, including all experimental protocols, was officially reviewed and approved by the Landesamt für Gesundheit und Soziales Berlin under the approval and license number G150/20, and followed the Laboratory Animal—Guideline for Ethical Review of Animal Welfare. Male New Zealand White rabbits (2.5–4 kg; Charles River Laboratories) were acclimatized in environmentally controlled laminar flow rooms with *ad libitum* access to food and water. To establish tumor models, VX2 tissue fragments were first inoculated into the quadriceps muscle of donor rabbits and grown over four weeks. Subsequently, harvested tumor material (0.3–0.5 mL) was transplanted into the left hepatic lobe of recipient animals via median laparotomy employing an 18‐gauge catheter. Surgical closures of the abdominal wall and skin were performed in two layers using absorbable sutures (chromic gut 3–0; Vicryl 4–0). Following a 14‐day growth period, MRI confirmed the formation of a solitary, well‐delineated tumor mass measuring 1.5–2 cm in diameter. Animals were allocated into three experimental groups: an untreated tumor control group (Day 0, D0; *n* = 5), a subacute group (Day 3, D3; *n* = 7) treated with incomplete microwave ablation (iMWA) and followed up for 3 days, and a long‐term group (Day 14, D14; *n* = 5) treated with iMWA and followed up for 14 days. The iMWA procedure was carried out with a 17F microwave needle (NeuWave Medical, Inc.) centrally placed along the longitudinal axis of the target tumor. The needle was stabilized by freezing to below −50 °C, and its positioning was assessed and confirmed using CT. Microwave energy was applied at 65 W for a duration of 40–60 s, aiming to achieve an ablation zone that included no less than 50% of the tumor. According to the generator readings, the temperature in the immediate vicinity of the needle tip reached 100–103 °C [15]. Liver tissues were collected following sacrifice at each respective time point. All operative procedures were performed under isoflurane anesthesia (1.5%–3%), with continuous monitoring of vital signs and thermal support provided to prevent hypothermia. Eventually, all samples were subsequently processed for histological staining with hematoxylin and eosin (H&E) and mass spectrometry‐based proteomic profiling.

### 2.2 Hematoxylin–eosin (HE) staining

Dissected tissues were rinsed with physiological saline and fixed in neutral formalin for 30–50 min. Afterward, they were washed in water three times for 5 min each, then dehydrated through graded ethanol solutions (70%, 80%, 90%) for about 30 min each, followed by two immersions in 95% and 100% ethanol for 20 min each. The tissues were then treated with an equal mixture of absolute ethanol and xylene for 15 min, cleared with xylene I and II for 15 min each until transparent, and infiltrated with a xylene‐paraffin mixture for 15 min. They were then embedded in paraffin and infiltrated at 55 °C–60 °C for 50–60 min in a constant temperature oven. After infiltration, the paraffin blocks were briefly warmed, poured into molds, and cooled. The blocks were sectioned into 5 μm slices with a microtome, floated on heated water, mounted onto slides, and dried at 45 °C. For HE staining, slides were deparaffinized in xylene, rehydrated through ethanol series (100% to 70%), then rinsed in water. Sections were stained with hematoxylin (X903.2; Roth) for 10–30 min, rinsed until nuclei turned blue, then briefly immersed in 1% hydrochloric acid ethanol to differentiate until tissues turned red or light. After washing to restore blue, they were counterstained with eosin (X883.2; Roth), Roth in graded ethanol (50%–80%), rinsed in ethanol, dehydrated, cleared in xylene, and coverslipped. This protocol ensures clear visualization of tissue morphology under the microscope.

### 2.3 Sample preparation

Paraffin‐embedded tissue blocks were sectioned into 10–20 μm slices. With reference to corresponding hematoxylin and eosin (H&E)‐stained sections, target regions of interest were carefully microdissected to minimize paraffin inclusion, and the isolated tissues were collected into prelabeled 1.5‐mL Eppendorf tubes. Each tube contained tissue from the same sample, with a total weight exceeding 3 mg based on empirical validation. For subsequent weighing, a specialized 8‐tube Covaris rack was used. After tarring the empty rack, tissue aliquots from each Eppendorf tube were sequentially transferred to achieve a target mass of 1.5 mg (range: 1–2 mg) per sample, with the balance tared between each addition. To expedite future proteomic workflows and reduce cross‐tissue contamination, it is advisable to separate distinct tissue types during the initial sample collection and embedding stages.

### 2.4 Proteomic determination

Global proteomic profiling was performed using a SCIEX ZenoTOF 7600 system coupled online to a Waters ACQUITY UPLC M‐Class system. Prior to MS analysis, 200 ng of incubated peptides was chromatographically separated using a 27‐min gradient at a flow rate of 5 μL/min on a Waters HSS T3 column (300 μm × 150 mm, 1.8 μm particle size) maintained at 35°C. Mobile phase A consisted of 0.1% formic acid (FA) in water, while mobile phase B consisted of 0.1% FA in acetonitrile. The gradient was programmed as follows: 1% to 40% B over 19 min, followed by an increase to 80% B over 1 min for column washing. The column was maintained at 80% B for 0.5 min and subsequently re‐equilibrated to the starting conditions for 6.5 min. A ZenoSWATH MS/MS acquisition scheme with an accumulation time of 11 ms and 85 variable‐width windows was used. The ion source parameters were set as follows: ion source gas 1, 12 psi; ion source gas 2, 60 psi; curtain gas, 25 psi; collision‐activated dissociation (CAD) gas, 7 psi; source temperature, 150°C; and spray voltage, 4500 V. Raw data were processed using DIA‐NN (version 1.8) with default settings. The fragment ion *m*/*z* range was set to 100–1800 *m*/*z*, and mass accuracy was set to 20 ppm for both MS1 and MS2. The scan window was set to 7, and match‐between‐runs (MBR) was enabled. Quantification was performed using the “Robust LC (high Precision)” strategy. A spectral‐library‐free approach was used for peptide identification and quantification, using the Oryctolagus cuniculus UniProt reference proteome (UP000001811), downloaded on March 7, 2024, as the sequence database. The resulting identifications were filtered at a 1% false discovery rate (FDR) at the peptide level.

### 2.5 Proteomic data analysis

Our proteomic determination identified a total of 8725 proteins. To assess differential protein expression, intensity values from all samples were normalized and subjected to logarithmic transformation to enhance comparability and stabilize data variance. The mean log2‐transformed expression level for each protein was calculated independently for the two comparison groups. The log2FC was derived as the arithmetic difference between these group means. Statistical evaluation was conducted using an empirical Bayes approach. The resulting log2FC values represent both the magnitude and direction of expression differences between the experimental groups. The investigation was initiated by cross‐referencing our dataset with a genomics database from our collaborative partner [16], which highlighted tumorigenic and immunologic genes elevated in a mouse RFA model. Pathway analysis of these proteins using STRING revealed a significant enrichment in tumor growth and immune response‐related pathways. To ensure a comprehensive assessment, we subsequently screened our entire proteomic pool for all additional constituents of these identified pathways. This systematic approach expanded our candidate list to include a broader set of relevant proteins. Finally, this refined dataset was subjected to an in‐depth bioinformatic interrogation using STRING and Enrichr.

### 2.6 Protein–protein interaction (PPI) and pathway analysis

PPI networks were constructed and analyzed using the STRING database (version 11.5; https://string‐db.org/). The network was generated with the following parameters: the medium confidence interaction score cutoff was set to 0.400 for network inclusion. To identify functionally coherent protein modules, the Markov Cluster Algorithm (MCL) was applied with an inflation parameter set to 3, which favors the identification of fine‐grained clusters. Only clusters containing a minimum of three protein‐coding genes were retained for further investigation. Functional enrichment analysis for the resulting PPI network was performed to identify overrepresented biological themes. Functional enrichment was performed for clusters containing ≥3 proteins, retaining relevant pathways with a false discovery rate (FDR) < 0.05. To gain deeper insights into the signaling pathways altered in our experimental model, we performed a comprehensive functional enrichment analysis of the differentially expressed proteins using Enrichr (https://maayanlab.cloud/Enrichr). Queries were run against three specialized knowledge bases: Reactome 2024, MSigDB Hallmark 2020, and WikiPathways 2024 Human. For all databases, enriched terms with an adjusted *P*‐value (FDR) < 0.05 were deemed statistically significant.

### 2.7 Stouffer analysis

Pathway‐level activation was further quantified using a curated 6‐gene Stouffer framework. Only proteins with a defined fold‐change and *P*‐value were included in Stouffer analysis. Canonical gene sets of 4–6 genes for each of 10 biological processes associated with liver ablation were applied here to the VX2 proteome (Table S7). In addition, two liver‐specific processes, ‘Complement/Coagulation’ and ‘Acute‐phase/Liver injury’, were defined *a priori* based on classical hepatocyte acute‐phase proteins. For each detected protein, *P*‐values were converted to two‐tailed z‐scores and assigned a sign according to the log_2_(FC). Missing canonical proteins were excluded from aggregation. Process‐level activation was computed using Stouffer's Z, defined as the sum of signed z‐scores divided by the square root of the number of detected canonical genes. Positive Z indicates pathway activation and negative Z indicates suppression. The resulting Day‐3 and Day‐14 process scores were summarized in a two‐column heatmap.

## 3 Results

### 3.1 Tissue characterization and proteomic profiling of liver tumors following incomplete microwave ablation (iMWA)

Partial ablation of targeted tumors was successfully induced. Based on gross examination including assessment of the fresh tissue color and texture, specimens were divided into the tumor ablation zone (indicated by the red arrows), areas of residual viable tumor tissue resulting from incomplete ablation (marked by the blue arrows), and the adjacent hepatic tissue (highlighted by the green arrows) (Fig. 1B). Critically, all subsequent proteomic analyses at both D3 and D14 were specifically performed on tissue samples microdissected from the identified regions of residual viable tumor at the ablation margin. H&E staining visualized and confirmed the spatial location of these tissue regions (Fig. 1C). The necrotic zone, characterized by a loss of cellular morphology, was clearly visible in the ablated tumor tissues with complete necrosis. By contrast, regions of viable tumor tissue retained intact cellular morphology; adjacent to these areas, edematous liver tissue was observed.

### 3.2 Proteomic profiling identified temporal dynamic protein expression in residual tumors postablation

**Fig. 2:**
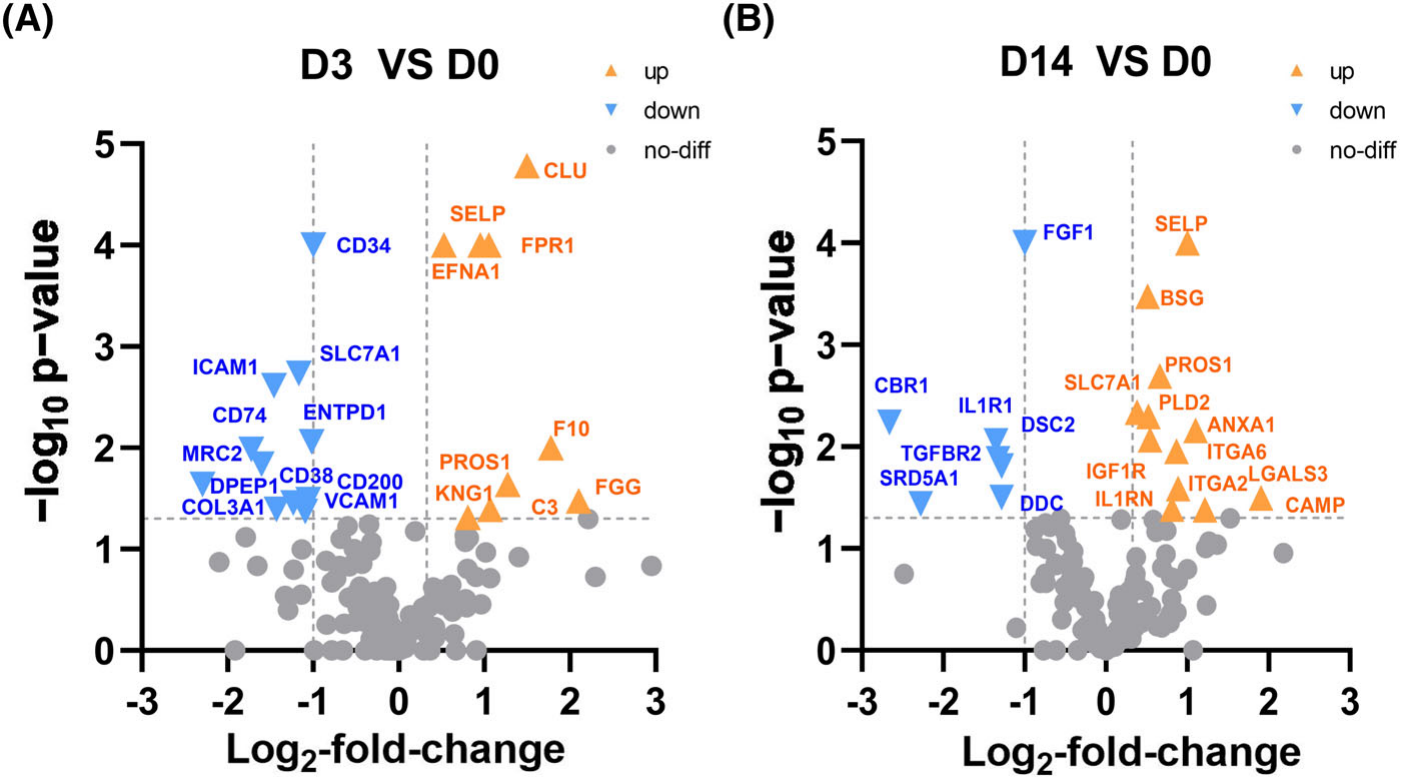
Volcano plot for significantly up‐ and down‐regulated proteins. Volcano plot depicting differentially expressed proteins. The y‐axis shows −log10 (*P*‐value), and the x‐axis displays log2 (ratio). Upregulated proteins are indicated by orange‐yellow dots, while down‐regulated proteins are represented by blue dots. Panel (A) corresponds to the D3 (*n* = 7) *vs*. D0 (*n* = 5) groups, and panel (B) illustrates the D14 (*n* = 5) *vs*. D0 (*n* = 5) groups. Statistical significance was determined using two‐tailed unpaired Welch's *t*‐test. The dashed horizontal line indicates *P* = 0.05.

Proteomics was performed for the untreated tissue from the D0 group (control tumors) and viable residual tumor from the D3 and from the D14 group. Proteomic profiling quantified 8724 proteins, with 240 overlapping transcriptomic hits. Among the 240 proteins, nine proteins were upregulated (FC >1.25, *P* < 0.05) and 11 down‐regulated (FC <0.50, *P* < 0.05) at the D3 group versus baseline (Fig. 2A). By D14, 12 proteins were significantly elevated and seven reduced (Fig. 2B). To further characterize the expression patterns of these proteins across the three groups, heatmaps of these differentially expressed proteins were generated (Fig. S1). Subsequently, the 20 upregulated proteins at D3 and 25 upregulated proteins at D14 were separately analyzed using STRING to construct protein–protein interaction networks. Network clusters enriched in pathways related to tumor growth and immune response were identified as pathways of interest. For these, we then mapped their constituent proteins with the full proteomic dataset of 8724 quantified proteins. This resulted in our ultimately identifying 81 upregulated molecules at D3 and 92 elevated ones at D14 (Table S1).

### 3.3 Protein clustering revealed structural reconstitution at D3

**Fig. 3:**
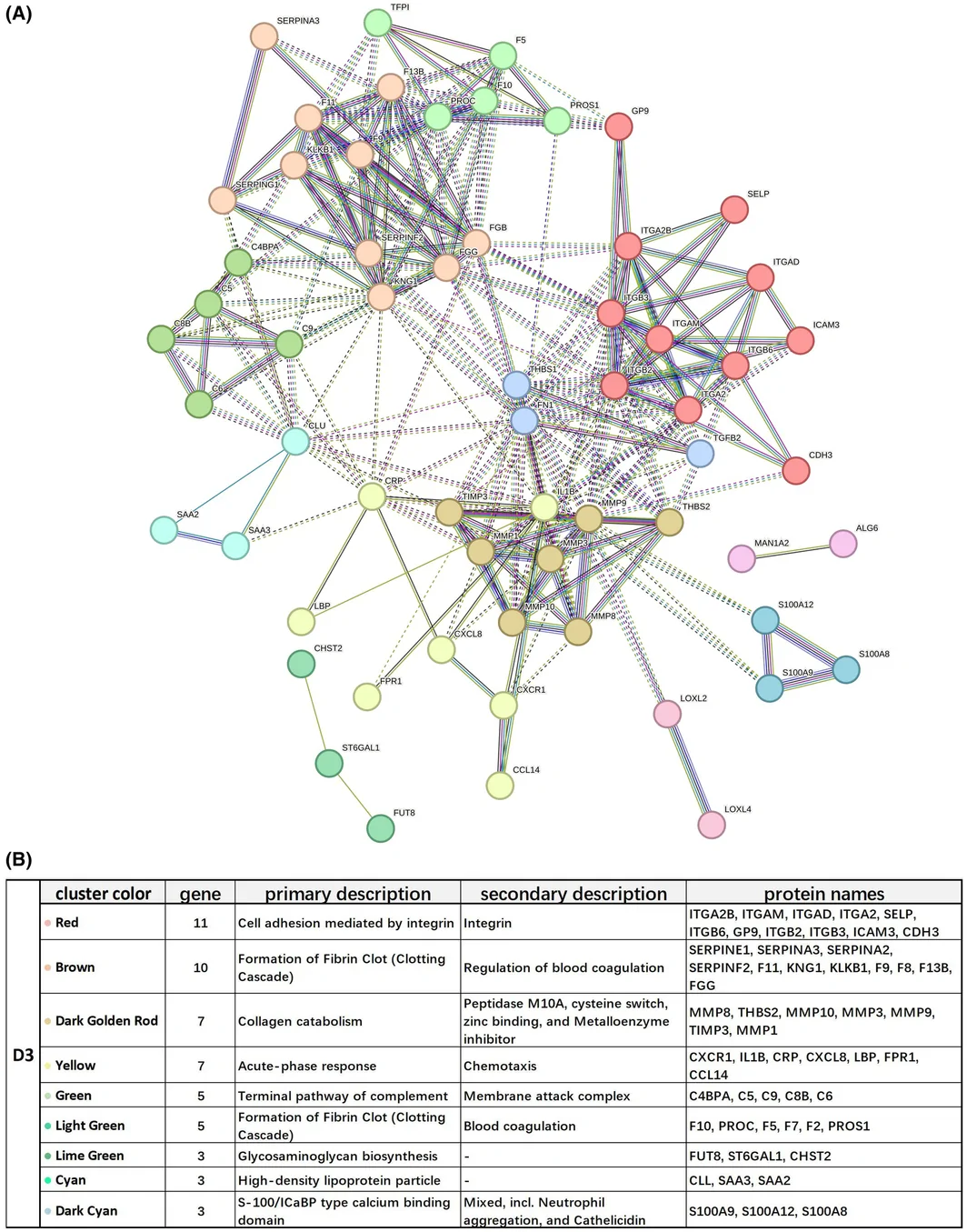
Network of upregulated proteins for D3. Protein–protein interaction (PPI) analysis was performed using STRING‐db with 81 upregulated proteins at D3 (A). The detailed information of these clusters with ≥ 3 counts presented in Fig. 4A was displayed in the table below. Each cluster is color‐coded and includes information on gene count, primary and secondary descriptions, and associated protein names (B). For more detailed information, please refer to https://string‐db.org/cgi/network?taskId=bNWrdwLwRht9&sessionId=bWlsEVoUzT7P.

At D3, nine protein clusters (comprising at least three genes) were identified (Fig. 3A), characterized by a predominant cell adhesion mediated by integrin cluster (*n* = 11). Functional annotation of these protein clusters highlighted strong involvement in acute injury response and early repair processes. Key clusters included proteins with complement and coagulation cascade activity (*n* = 12), MHC class II‐mediated antigen presentation (*n* = 9), and lysosomal cathepsin and protein degradation (*n* = 8). Additional clusters were associated with lipid metabolism and transport proteins (*n* = 7), inflammatory signal mediators and calcium‐binding proteins (*n* = 6), and cytoplasmic structural proteins (*n* = 5) (Fig. 3B). Gene Ontology (GO) enrichment analysis further supported these findings, revealing significant enrichment in terms such as ‘extracellular matrix’ (*n* = 15, FDR = 4.28e‐09) and ‘collagen catabolic process’ (*n* = 5, FDR = 3.41e‐05). Reactome pathway analysis also identified ‘ECM proteoglycans’ (*n* = 5, FDR = 0.00015) and ‘Elastic fiber formation’ (*n* = 5, FDR = 7.96e‐06) as highly represented (Table S2).

### 3.4 Protein clustering revealed tumor growth and immune regulation at **D14** group

**Fig. 4:**
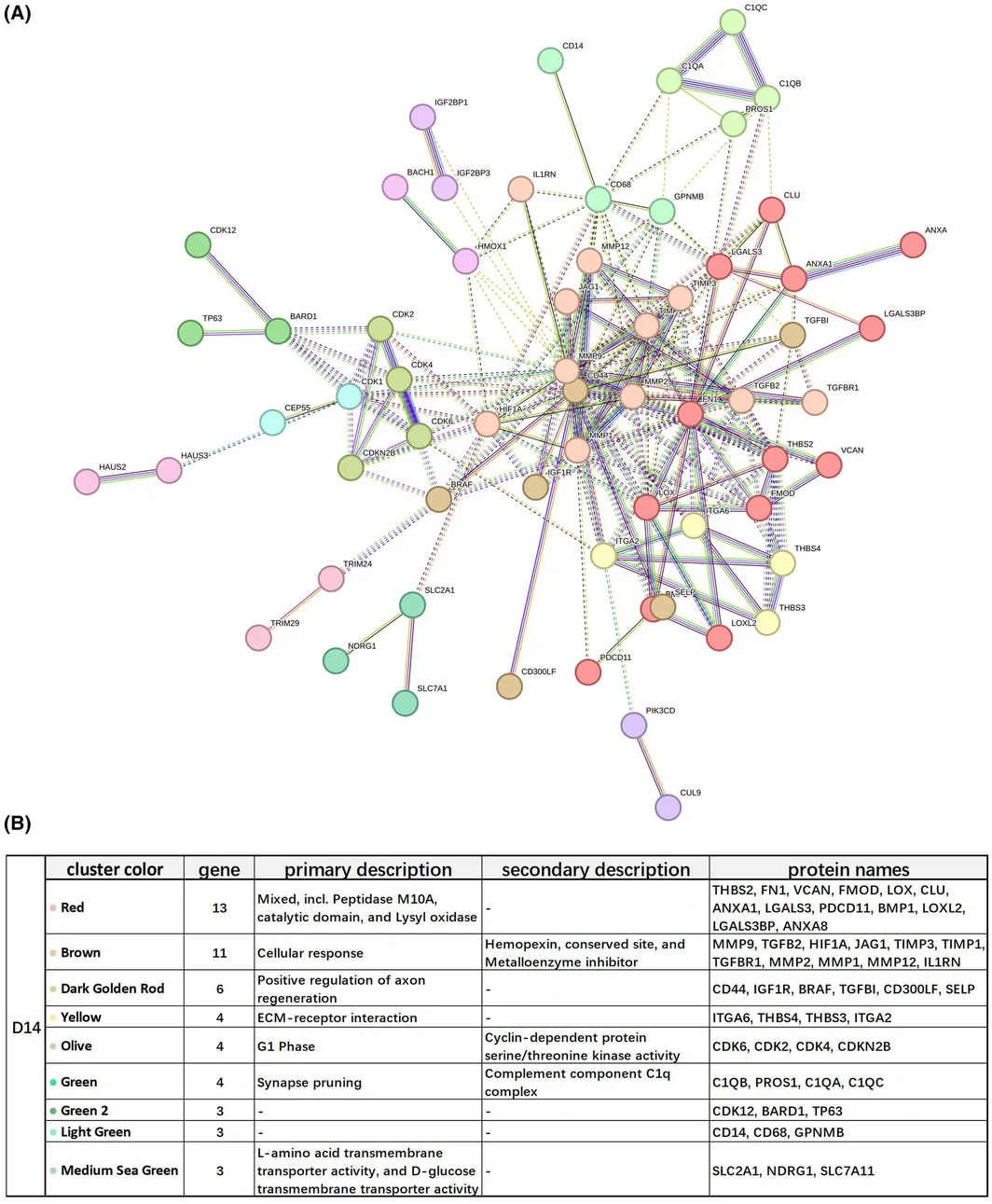
Network of the upregulated proteins for D14. PPI analysis was performed using STRING‐db with 92 upregulated proteins at D14 (A). The detailed information of these clusters with ≥3 counts presented in Fig. 5A was displayed in the table below. Each cluster is color‐coded and includes information on gene count, primary and secondary descriptions, and associated protein names (B). For more detailed information, please refer to https://string‐db.org/cgi/network?taskId=bCIujZvnMoNa&sessionId=bWlsEVoUzT7P.

At D14, a total of eight functional clusters comprising at least three genes were characterized within the proteomic profile (Fig. 4A). These included clusters functionally annotated as mixed, incl. Peptidase M10A, catalytic domain, and Lysyl oxidase (*n* = 13), cellular response (*n* = 11), positive regulation of axon regeneration (*n* = 6), and ECM‐receptor interaction (*n* = 4). Additionally, clusters related to the G1 Phase (*n* = 4) and synapse pruning (*n* = 4) were identified. Two further clusters containing at least three genes remained unannotated by the automated pipeline, which were designated as the DNA damage response cluster (consisting of CDK12, BARD1, and TP63; *n* = 3) and the macrophage phenotype cluster (composed of CD14, CD68, and GPNMB; *n* = 3) (Fig. 4B). GO enrichment analysis underscored this transition, highlighted by significant terms associated with cell proliferation and growth signaling. Specifically, these included ‘regulation of cell population proliferation’ (FDR = 9.64e‐12), ‘growth factor binding’ (FDR = 0.0021), and specific enzymatic activities such as ‘RNA polymerase II CTD heptapeptide repeat kinase activity’ (FDR = 0.00043) and ‘cyclin‐dependent protein serine/threonine kinase activity’ (FDR = 0.00043). Concurrently, pathways modulating cell fate and turnover were significantly enriched, notably ‘positive regulation of cell death’ (FDR = 8.42e‐07) and ‘positive regulation of apoptotic process’ (FDR = 1.56e‐06), reinforcing a coordinated shift toward active tumor growth signaling during the later stage (Table S3).

### 3.5 Functional enrichment analysis identifies temporal shifts in signaling pathways from early **ECM** remodeling to late immune and oncogenic signaling

To further delineate alterations in signaling pathways following ablation, we conducted functional enrichment analysis of upregulated proteins using Enrichr (https://maayanlab.cloud/Enrichr) with a focus on the Reactome 2024, MSigDB Hallmark 2020, and WikiPathways 2024 Human databases. Pathway analysis revealed a significant transition in the biological landscape from D3 to D14. At D3, the tissue microenvironment was dominated by pathways associated with acute injury response (*n* = 18), the complement system (*n* = 20), and ECM remodeling/fibrosis (*n* = 27). By D14, these early‐phase pathways underwent a marked decline, with their enrichment counts dropping to 6, 4, and 8, respectively. In contrast, at D14, it was characterized by a distinct shift toward complex signaling and proliferative activity. Signal transduction pathways exhibited a robust expansion, with the number of enriched pathways increasing from 13 at D3 to 35 at D14 (e.g., PI3K‐Akt signaling pathway, TGF‐beta signaling pathway). Furthermore, pathways related to tumorigenesis and immune function showed marked upregulation (Tumorigenesis: 5 to 11; Immune Function: 12 to 16). While pathways involved in angiogenesis showed a moderate increase from D3 (*n* = 1) to D14 (*n* = 6), the overall progression from acute inflammatory responses to complex signal transduction and enhanced immune‐tumorigenic signaling underscores the dynamic and multistage nature of tissue remodeling. (see Tables 1, S4 and S5).

**Table 1:** Comparison of biological pathway activation at D3 and D14.

Category	D3_count	D14_count	Change (D14 vs. D3)
ECM remodeling/fibrosis	27	8	−19
Acute injury response	18	6	−12
Complement system	20	4	−16
Signal transduction	13	35	23
Tumorigenesis	5	11	6
Immune function	12	16	4
Angiogenesis/vascular	1	6	5

*Note*: This table depicts the changes in pathway participation across the various pathway categories between D3 and D14.

### 3.6 Immune reprogramming analysis identifies the synergistic coupling of macrophage functional remodeling and immune checkpoint signaling

**Fig. 5:**
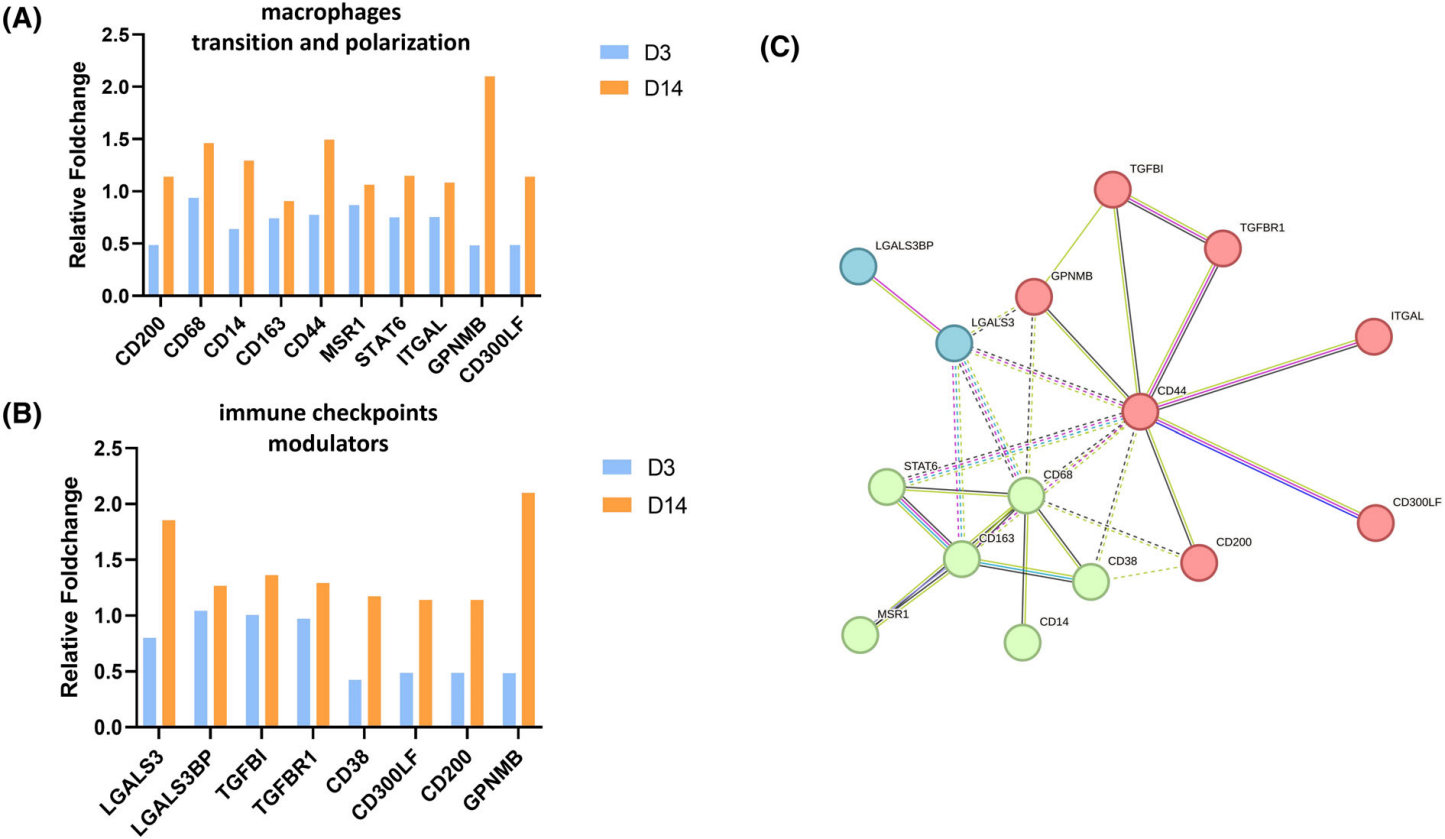
Temporal dynamics of macrophage activation and polarization and immune checkpoint signaling. The markers of macrophage transition and polarization were increased at D14 compared with D3 (A), and the immune checkpoint modulators were increased at D14 compared with D3 (B). PPI analysis was performed using STRING‐db with 15 upregulated proteins involved in macrophage transition, polarization, and immune checkpoint signaling at D14 compared with D3. For more detailed information, please refer to https://string‐db.org/cgi/network?taskId=bCPdkXChKPbi&sessionId=bIuqPRYMXMP4 (C). Values represent relative fold changes derived from expression data, illustrating temporal expression trends without additional statistical hypothesis testing.

Given the notable enrichment of macrophage‐associated markers—specifically CD14, CD68, and GPNMB—within the D14 cell cluster, we further investigated the immune characteristics dynamics from D3 to D14, and markers and modulators involved in macrophage transition, polarization, and immune checkpoint signaling were analyzed. The selection of specific protein markers was based on their well‐defined and established roles supported by the existing literature. Comparative analysis between D14 and D3 revealed a coordinated upregulation of genes associated with a transition toward an immunosuppressive macrophage phenotype, including CD200 [17], CD68 [18], CD14 [19], CD163 [20], CD44 [21], MSR1 [22], STAT6 [23], ITGAL [24], GPNMB [25], and CD300LF [26] (Table S6 and Fig. 5A). Concurrently, a significant induction of immune checkpoint modulators was observed, encompassing LGALS3 [27], LGALS3BP [28], TGFBI [29], TGFBR1 [30], CD38 [31], CD300LF [32], CD200 [33], and GPNMB [34] (Table S6 and Fig. 5B). To elucidate the functional integration of these molecules, we performed PPI network analysis via the STRING database. The analysis revealed a highly interconnected network in which CD44 emerged as a central hub, physically and functionally bridging the macrophage‐associated and immune checkpoint modules (Fig. 5C). Furthermore, GO enrichment analysis highlighted ‘negative regulation of immune system process’ (GO:0002683, FDR = 0.0266) as a key functional outcome of this network.

### 3.7 Stouffer analysis confirms the temporal shift from early matrix remodeling and cell death to late immune activation and resolution with immune suppression

Application of the canonical 6‐gene Stouffer framework (Table S7) to the VX2 liver proteome demonstrated significant shifts in functional pathways across the postablation timeline. Cell‐death and necro‐inflammatory programs, including pyroptosis and ferroptosis, remained elevated at both D3 (*Z* = 2.81) and D14 (*Z* = 2.45). In contrast, fibrogenic remodeling and EMT/progenitor activation pathways showed initial induction at D3 (*Z* = 1.78), followed by a decline to below baseline levels at D14 (*Z* = −1.22). The complement/coagulation and acute‐phase response pathways exhibited persistent and substantial suppression throughout the observation period, with *Z*‐scores of −7.62 at D3 and −6.55 at D14. Concurrently, metabolic reprogramming and ER stress pathways shifted from relative suppression at D3 (*Z* = −0.65) to activation at D14 (*Z* = 0.82).

**Fig. 6:**
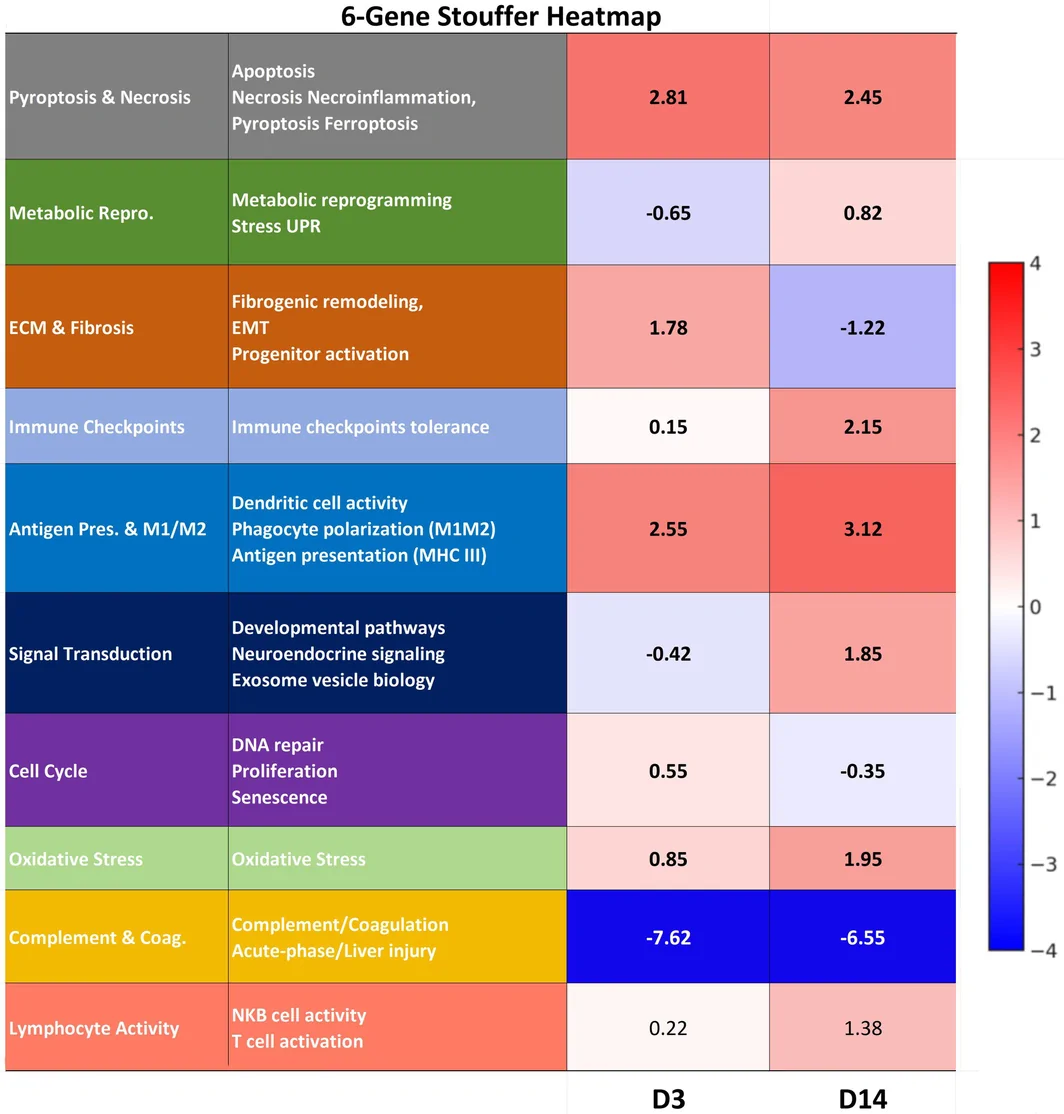
Canonical Stouffer Pathway Heatmap. The Canonical 6‐gene Stouffer heatmap summarizing pathway activation following VX2 liver ablation. Each row represents a biological process defined by curated 6‐gene canonical sets (Table S7), derived from proteins with defined fold‐change and *P*‐value. Color scale represents Stouffer Z‐score (blue = suppression, red = activation, white = neutral). Numerical values indicate the aggregated *Z*‐score for Day 3 and Day 14 relative to baseline, with pathway activation significance evaluated based on the standard normal distribution of Stouffer's *Z*‐scores.

Adaptive immune components also displayed temporal variations. Antigen presentation and dendritic cell signatures showed sustained elevation, rising from *Z* = 2.55 at D3 to *Z* = 3.12 at D14. Lymphocyte activity (including T‐cell and NK/NKT cell signatures) transitioned from near‐baseline levels at D3 (*Z* = 0.22) to increased activation at D14 (*Z* = 1.38). This was accompanied by a marked increase in immune checkpoint and tolerance‐related molecule expression, shifting from *Z* = 0.15 at D3 to *Z* = 2.15 at D14. Additionally, oxidative stress markers exhibited a more pronounced activation at D14 (*Z* = 1.95) compared to D3 (v = 0.85), while developmental and neuroendocrine signaling pathways also trended toward late‐stage activation (*Z* = 1.85 at D14). (Fig. 6).

## 4 Discussion

MWA has emerged as a minimally invasive treatment option for early‐stage liver cancer, offering reliable efficacy and a favorable safety profile. However, the molecular mechanisms driving the progression of residual tumors following incomplete ablation remain to be fully elucidated. By integrating a systemic whole‐liver genomic dataset [16] with our proteomic profiles, we propose a conceptual framework delineating a dynamic temporal evolution within the residual tumor. This progression is characterized by a distinctive transition from an early structural repair‐dominant phase (D3) to a subsequent immunosuppressive and signaling‐intensive phase (D14).

More precisely, at D3, the microenvironment of the residual tumor is dominated by an acute injury response and ECM remodeling. The marked upregulation of structural and adhesion molecules (FN1, FGG, FGB, ICAM3, LOXL2), alongside the activation of complement cascades and ECM‐related pathways such as collagen formation, elastic fiber assembly, and glycosaminoglycan biosynthesis establishes a provisional fibrotic scaffold [35]. While essential for initial wound healing, this provisional matrix is ultimately co‐opted by surviving tumor cells [36]. By D14, this reparative state undergoes a profound protumorigenic switch. Early ECM pathways precipitously decline, superseded by robust oncogenic signal transduction notably the PI3K‐Akt and TGF‐β pathways. The activation of PI3K‐Akt provides potent survival and proliferative cues for residual tumor cells [37], aligning with the observed enrichment in ‘regulation of cell population proliferation’. Concurrently, TGF‐β signaling acts as a double‐edged sword: while it drives late‐stage tissue remodeling, it is also a master regulator of immune suppression [38]. Parallel to this signaling shift, the local immune compartment is radically reprogrammed toward immunosuppression. Macrophages abandon early restorative functions for a tolerogenic phenotype, characterized by the synergistic coupling of functional markers (CD163, STAT6, GPNMB) with a comprehensive immune checkpoint network (LGALS3, TGFBI, CD200). Crucially, our PPI analysis identifies CD44 as the central regulatory hub orchestrating this crosstalk, bridging the macrophage and checkpoint modules to drive the negative regulation of immune system processes. Functionally, CD44 not only anchors these tolerogenic macrophages to the tumor stroma via ligands like hyaluronic acid, but also actively amplifies co‐inhibitory signaling [39], thereby restricting T‐cell activation and consolidating an immune‐privileged niche.

Critically, our data suggest that this early‐stage ECM remodeling may function as a priming event that helps orchestrate the subsequent development of an immunosuppressive microenvironment. The rapid deposition of specific collagens at D3 potentially establishes a physical and biochemical barrier that modulates immune cell infiltration [40]. For instance, the dense collagen scaffold could sequester protumorigenic growth factors and create a hypoxic niche, providing putative mechanotransduction signals that drive the functional transition from pro‐inflammatory responses toward the protumorigenic state [41, 42] observed by D14. From a translational perspective, the distinct biophysical and biochemical shifts identified in our study specifically the early deposition of collagen and elastin offer a unique diagnostic window for non‐invasive monitoring. These structural changes significantly alter tissue stiffness and composition at the ablation margin, which can be quantified using advanced imaging modalities such as Magnetic Resonance Elastography (MRE) and elastin‐specific molecular probes [43, 44]. Recent studies have demonstrated that MRE can effectively map tissue visco‐elasticity to characterize the tumor microenvironment [45], while elastin‐targeted imaging provides high‐resolution insights into extracellular matrix remodeling [46]. Integrating these imaging biomarkers into postablation surveillance protocols could enable the early, noninvasive identification of ‘pro‐residual tumor growth’ niches, allowing for personalized intervention before macroscopic tumor regrowth occurs. While further functional studies are required to definitively link these temporal phases, these identified candidates, particularly the core nodes in our network (e.g., CD44 and the PI3K‐Akt axis), represent the most logical targets for future functional perturbation assays to further validate their causative roles in postablation residual tumor growth.

To improve curative outcomes, current HCC management increasingly favors the combination of locoregional therapies with systemic agents targeting residual tumor cells or the postablation microenvironment [47]. Our findings provide a strong mechanistic rationale for the selection and precise timing of such adjuvant strategies. Specifically, our proteomic and enrichment analyses revealed a pronounced shift toward active tumor growth at D14, marked by the significant activation of cell cycle pathways and the concurrent upregulation of key cyclin‐dependent kinases (CDK1, CDK2, CDK4, CDK6, and CDK12). Given this robust proliferative signature, the targeted use of CDK inhibitors could effectively arrest cell cycle progression and halt the early expansion of residual tumors [48]. Importantly, cell cycle inhibitors have been shown to synergize with existing HCC standards, including systemic agents such as lenvatinib [49] and radiotherapy [50]. Building on this established functional synergy, integrating CDK inhibitors with MWA presents a highly promising approach to suppress residual tumor growth. Furthermore, our network analysis identified CD44 as a central hub orchestrating an immunosuppressive niche. By bridging tolerogenic macrophages with an extensive immune checkpoint network, CD44 facilitates immune evasion postablation. The therapeutic potential of this axis is supported by emerging evidence, where CD44‐targeted delivery platforms have demonstrated remarkable synergy with local embolization [51].

When interpreted alongside the macrophage and immune checkpoint modulator panels, the canonical Stouffer analysis supports a model of postablation ‘immune paralysis’ rather than robust antitumor immunity. Marker‐level data show a pronounced protumorigenic macrophage shift and increased immune evasion markers, indicating functional suppression of both innate and adaptive arms [52]. The Stouffer framework complements this by demonstrating a dramatic collapse of complement and hepatocyte acute‐phase responses, alongside sustained pyroptotic/ferroptotic injury signatures. Although antigen presentation and T‐cell activation pathways are elevated between D3 and D14, the simultaneous loss of effector functions and the M2‐skewed, checkpoint‐poor yet functionally suggest that this activation is compensatory rather than curative. This reconciled view identifies a mechanistically rational window for intervention during the D3–D14 interval, where augmenting innate danger signaling and relieving T‐cell suppression may shift the landscape toward productive immunity. Clinical trials support this rationale: adding ablation to anti‐PD‐1 therapy increased response rates [53]. Notably, the IMbrave050 study demonstrated that adjuvant atezolizumab plus bevacizumab significantly improved recurrence‐free survival for ablated high‐risk HCC patients [54], while cellular therapies, such as mRNA‐loaded dendritic cell vaccines, further offer potential in reducing postablation residual tumor growth [55].

Several limitations should be considered when interpreting these results. First, this study integrated proteomic data from a rabbit tumor model of MWA with transcriptomic data from a mouse model that employed hepatic RFA. While interspecies and modality differences are likely to influence the precise cellular response, both models capture the shared biological framework of thermal injury and tissue repair, allowing meaningful integration at the pathway level and cross‐validation of general post‐ablation processes. Indeed, this is the biologic rationale of the vast majority of mouse studies to elucidate human biology. Second, differences in tissue type and sampling timeline between these two studies limit the direct comparison. We readily note that murine genomic data were obtained from normal liver tissue at Days 1 and 7 postablation, whereas rabbit proteomic data were derived from tumor tissue at Days 3 and 14. To address this, we focused on conserved temporal trends and overlapping pathways, which consistently highlighted ECM remodeling, immune regulation, and oncogenic signaling as central postablation processes. Third, the VX2 tumor model originates from rabbit squamous carcinoma and not HCC or adenocarcinomas such as colorectal subtypes typically treated in clinical practice. Nevertheless, it was chosen for its hypervascularity and imaging resemblance to human HCC, and a similar hyperglycolytic phenotype, making it a well‐established translational model for ablation research in liver cancer [56]. Moreover, the model provided many benefits relevant to this study, including the immunocompetence of the model, the solitary and reproducible tumor growth, and the animal size that allows for use with human ablation probes and imaging equipment and thus, facilitates clinical translation. Additionally, because the rabbit proteome is incompletely annotated and lacks reviewed canonical sequences, we performed peptide identification against the well‐curated mouse UniProtKB reference. Although this cross‐species approach is widely accepted in translational proteomics and prioritizes high‐confidence identification and pathway assignment, nevertheless, species‐specific isoforms and VX2 lineage variations may be underrepresented, and results should therefore be interpreted as orthology‐based approximations. Finally, pathway annotation relied on human‐based databases due to the absence of rabbit‐specific resources; this cross‐species approach, while imperfect, is widely accepted in translational proteomics and captures conserved signaling networks relevant to human liver cancer biology [57].

## 5 Conclusion

In conclusion, this study provides molecular insights into signaling and pathways related to liver tumor residual tumor growth after incomplete ablation. The findings highlight a biphasic postablation response comprising early ECM‐driven repair and oncogenic signaling at 3 days followed by a picture of relative immune suppression by Day 14 postablation. Thus, our data outline a temporal transition from structural recovery to tumor‐promoting reprogramming that likely underpins early residual tumor growth. Understanding these sequential adaptive processes provides a mechanistic framework for optimizing postablation therapy. Integrating MWA with targeted kinase‐inhibitory or immunomodulatory strategies holds promise for disrupting residual tumor growth‐promoting pathways and improving long‐term clinical outcomes in liver cancer.

## Conflict of interest

The authors declare no conflict of interest.

## Author contributions

LJS and SNG conceptualized the project. LJS, SNG, MS, YH, and YL designed and optimized the experiments. YL, YH, NB, MM, KTT, JG, and JR performed the experiments and data analysis. YL, JR, YH, LJS, and SNG contributed to the revision work. YL wrote the manuscript. All authors reviewed and revised the manuscript. LJS and SNG supervised the whole project.

## Supporting information


**Figure S1.** Heatmap of differentially expressed proteins across D0, D3, and D14 groups.
**Table S1.** List of upregulated proteins at D3 and D14.
**Table S2.** List of the functional enrichments of protein network for D3 group.
**Table S3.** List of the functional enrichments of protein network for D14 group.
**Table S4.** Enriched pathways of upregulated proteins at D3.
**Table S5.** Enriched pathways of upregulated proteins at D14.
**Table S6.** Fold changes of markers/regulators for macrophages and immune checkpoints.
**Table S7.** Canonical gene sets used for stouffer analysis.

## Data Availability

Reasonable requests for the data and materials supporting this article will be fulfilled by the corresponding author (lynn-jeanette.savic@charite.de).

## References

[1] GBD 2023 Cancer Collaborators . The global, regional, and national burden of cancer, 1990–2023, with forecasts to 2050: a systematic analysis for the global burden of disease study 2023. Lancet. 2025;25:S0140. 10.1016/S0140-6736(25)01635-6 PMC12687902 41015051

[2] European Association for the Study of the Liver . EASL clinical practice guidelines on the management of hepatocellular carcinoma. J Hepatol. 2025;82(2):315–374. 10.1016/j.jhep.2024.08.028 39690085

[3] Reig M , Sanduzzi‐Zamparelli M , Forner A , Rimola J , Ferrer‐Fàbrega J , Burrel M , et al. BCLC strategy for prognosis prediction and treatment recommendations: the 2026 update. J Hepatol. 2026;84(3):631654. 10.1016/j.jhep.2025.10.020 41151697

[4] van der Lei, S , Puijk, RS , Dijkstra, M , Schulz, HH , Vos, DJ , De Vries, JJ , et al. Thermal ablation versus surgical resection of small‐size colorectal liver metastases (COLLISION): an international, randomised, controlled, phase 3 non‐inferiority trial. Lancet Oncol. 2025;26:187–199. 10.1016/S1470-2045(24)00660-0 39848272

[5] Yamauchi Y , Sakao Y . Ablation treatment for peripheral lung cancers. Kyobu Geka. 2025;78:871–876. 40998355

[6] Sugimoto K , Imajo K , Kuroda H , Murohisa G , Shiozawa K , Sakamaki K , et al. Microwave ablation vs. single‐needle radiofrequency ablation for the treatment of HCC up to 4 cm: a randomized‐controlled trial. JHEP Rep. 2024;13(7):101269. 10.1016/j.jhepr.2024.101269 PMC11719385 39802807

[7] Dong F , Wu Y , Li W , Li X , Zhou J , Wang B , et al. Advancements in microwave ablation for tumor treatment and future directions. iScience. 2025;7(28):112175. 10.1016/j.isci.2025.112175 PMC12017980 40271529

[8] Pillai K , Akhter J , Chua TC , Shehata M , Alzahrani N , Al‐Alem I , et al. Heat sink effect on tumor ablation characteristics as observed in monopolar radiofrequency, bipolar radiofrequency, and microwave, using an ex vivo calf liver model. Medicine (Baltimore). 2015;94(9):e580. 10.1097/MD.0000000000000580 PMC4553952 25738477

[9] Radosevic A , Quesada R , Serlavos C , Sánchez J , Zugazaga A , Sierra A , et al. Microwave versus radiofrequency ablation for the treatment of liver malignancies: a randomized controlled phase 2 trial. Sci Rep. 2022;12(1):316. 10.1038/s41598-021-03802-x PMC8748896 35013377

[10] Zhang NN , Lu W , Cheng XJ , Liu JY , Zhou YH , Li F . Clinical efficacy of high‐power microwave ablation in the treatment of hepatocellular carcinoma and analysis of risk factors for recurrence. J Pract Hepatol. 2015;18:249–253.

[11] Kotoh K , Nakamuta M , Morizono S , Kohjima M , Arimura E , Fukushima M , et al. A multi‐step, incremental expansion method for radio frequency ablation: optimization of the procedure to prevent increases in intra‐tumor pressure and to reduce the ablation time. Liver Int. 2005;25:542–547. 10.1111/j.1478-3231.2005.01051.x 15910491

[12] Ahmed M , Kumar G , Moussa M , Wang Y , Rozenblum N , Galun E , et al. Hepatic radiofrequency ablation‐induced stimulation of distant tumor growth is suppressed by c‐met inhibition. Radiology. 2016;279:103–117. 10.1148/radiol.2015150080 PMC4819900 26418615

[13] Forner A , Reig M , Bruix J . Hepatocellular carcinoma. Lancet. 2018;391:1301–1314. 10.1016/S0140-6736(18)30010-2 29307467

[14] Shi ZR , Duan YX , Cui F , Wu ZJ , Li MP , Song PP , et al. Integrated proteogenomic characterization reveals an imbalanced hepatocellular carcinoma microenvironment after incomplete radiofrequency ablation. J Exp Clin Cancer Res. 2023;42(1):133. 10.1186/s13046-023-02716-y PMC10210354 37231509

[15] He Y , Xu H , Rogasch J , Berndt N , Guo J , Schellenberger E , et al. Molecular MRI of tumor‐associated macrophages to monitor immunosuppression after incomplete ablation in liver cancer. Adv Sci. 2026; (Under revision).

[16] Stechele M , Amadi J , Salvermoser L , Sperlich L , Monin J , Khademi D , et al. Hepatic radiofrequency ablation induces global cellular activation throughout the liver. Eur Radiol Exp. 2026;10(1):38. 10.1186/s41747-026-00687-1 PMC13047045 41926011

[17] Li J , Wang Z , Qin X , Zhong MC , Tang Z , Qian J , et al. CD200R1‐CD200 checkpoint inhibits phagocytosis differently from SIRPα‐CD47 to suppress tumor growth. Nat Commun. 2025;16:5145. 10.1038/s41467-025-60456-3 PMC12134331 40461553

[18] Muntinga CLP , van de Sande AJ , Welters MJP , Kooreman LFS , Bekkers RLM , van Beekhuizen H , et al. Local immunosuppressive microenvironment underlies reduced responsiveness to imiquimod treatment in recurrent or residual cervical HSIL. Gynecol Oncol Rep. 2026;65:102121. 10.1016/j.gore.2026.102121 PMC13241649 42253310

[19] Quero L , Hanser E , Manigold T , Tiaden AN , Kyburz D . TLR2 stimulation impairs anti‐inflammatory activity of M2‐like macrophages, generating a chimeric M1/M2 phenotype. Arthritis Res Ther. 2017;19(1):245. 10.1186/s13075-017-1447-1 PMC5667453 29096690

[20] Liu Y , Chen S , Xu J , Yuan H , Yang H , Deng B , et al. Macrophage‐derived CTSZ promotes prostate cancer progression via αVβ3 integrin‐mediated activation of the AKT/FOXO1/JUNB signaling axis. Cancer Cell Int. 2026; Jun; Online ahead of print. 10.1186/s12935-026-04392-2 PMC13560195 42351165

[21] Yang J , Yin W , Ge J , Gao G , Yao X , Huang Y . Thyroid cancer‐derived exosomal SPP1 promotes tumor progression by driving macrophage M2 polarization through the CD44/JAK2/STAT3 signaling pathway. Organogenesis. 2026;22(1):2670152. 10.1080/15476278.2026.2670152 PMC13192093 42153613

[22] Takashi I , Kanai R , Seki M , Agata H , Kagami H , Murata H , et al. Inhibition of the TLR4/RAGE pathway by clearance of extracellular HMGB1 is a potential therapeutic target for radiation‐damaged salivary glands. Regen Ther. 2025;30:476–490. 10.1016/j.reth.2025.07.004 PMC12335976 40791981

[23] Jiang Z , Zhang X , Jin L , Han M , Zhang Y , Jiang Y , et al. Innovative insights into interleukin‐mediated macrophage polarization: metabolic reprogramming and inflammatory pathway crosstalk in chronic kidney disease and therapeutic implications‐a narrative review. Int J Gen Med. 2026;19:610534. 10.2147/IJGM.S610534 PMC13281874 42325941

[24] Chen F , Bai G , Liu Q , He G , Ding Z , Liang J , et al. Integrated multi‐omics identifies a CD54+ iCAF‐ITGAL+ macrophage niche driving immunosuppression via CXCL8‐PDL1 axis in cervical cancer. Mol Cancer. 2025;24(1):262. 10.1186/s12943-025-02471-y PMC12539134 41121145

[25] Lin Y , Qi Y , Jiang M . Lactic acid‐induced M2‐like macrophages facilitate tumor cell migration and invasion via the GPNMB/CD44 axis in oral squamous cell carcinoma. Int Immunopharmacol. 2023;124:110972. 10.1016/j.intimp.2023.110972 37806107

[26] Sutherland SIM , Ju X , Silveira PA , Kupresanin F , Horvath LG , Clark GJ . CD300f signalling induces inhibitory human monocytes/macrophages. Cell Immunol. 2023;390:104731. 10.1016/j.cellimm.2023.104731 37302321

[27] Ren Y , Qian Y , Zhang Q , Li X , Li M , Li W , et al. High LGALS3 expression induced by HCP5/hsa‐miR‐27b‐3p correlates with poor prognosis and tumor immune infiltration in hepatocellular carcinoma. Cancer Cell Int. 2024;24(1):142. 10.1186/s12935-024-03309-1 PMC11031979 38643145

[28] Wang C , Chen J , Wang W . Prognostic significance of LGALS3BP in triple‐negative breast cancer: implications for immune infiltration and therapeutic response. J Biochem Mol Toxicol. 2026;40(1):e70536. 10.1002/jbt.70536 41467329

[29] Zou S , Li L , Lv B . TGFBI inhibits the Pyroptosis of macrophages to ameliorate septic shock. J Cell Mol Med. 2025;29(19):e70802. 10.1111/jcmm.70802 PMC12516155 41078093

[30] Lei Z , Tang R , Wu Y , Mao C , Xue W , Shen J , et al. TGF‐β1 induces PD‐1 expression in macrophages through SMAD3/STAT3 cooperative signaling in chronic inflammation. JCI Insight. 2024;9(7):e165544. 10.1172/jci.insight.165544 PMC11128204 38441961

[31] Chen L , Diao L , Yang Y , Yi X , Rodriguez BL , Li Y , et al. CD38‐mediated immunosuppression as a mechanism of tumor cell escape from PD‐1/PD‐L1 blockade. Cancer Discov. 2018;8(9):1156–1175. 10.1158/2159-8290.CD-17-1033 PMC6205194 30012853

[32] Wang C , Zheng X , Zhang J , Jiang X , Wang J , Li Y , et al. CD300ld on neutrophils is required for tumour‐driven immune suppression. Nature. 2023;621(7980):830–839. 10.1038/s41586-023-06511-9 37674079

[33] Moon SY , Han M , Ryu G , Shin SA , Lee JH , Lee CS . Emerging immune checkpoint molecules on cancer cells: CD24 and CD200. Int J Mol Sci. 2023;24(20):15072. 10.3390/ijms242015072 PMC10606340 37894750

[34] Chung JS , Ramani V , Guo L , Popat V , Cruz PD Jr , Xu L , et al. Acquired resistance to immune checkpoint therapy is caused by glycoprotein non‐metastatic melanoma protein B signal cascade. Commun Med. 2025;5(1):79. 10.1038/s43856-025-00786-x PMC11926377 40114009

[35] Park H , Patil TV , Dutta SD , Lee J , Ganguly K , Randhawa A , et al. Extracellular matrix‐bioinspired anisotropic topographical cues of electrospun nanofibers: a strategy of wound healing through macrophage polarization. Adv Healthc Mater. 2024;13(12):e2304114. 10.1002/adhm.202304114 38295299

[36] Kolesnikoff N , Chen CH , Samuel MS . Interrelationships between the extracellular matrix and the immune microenvironment that govern epithelial tumour progression. Clin Sci (Lond). 2022;136(5):361–377. 10.1042/CS20210679 PMC8907655 35260891

[37] Hotfilder M , Sondermann P , Senss A , Valen F , Jürgens H , Vormoor J . PI3K/AKT is involved in mediating survival signals that rescue Ewing tumour cells from fibroblast growth factor 2‐induced cell death. Br J Cancer. 2005;92(4):705–710. 10.1038/sj.bjc.6602384 PMC3216036 15685229

[38] van den Bulk J , de Miranda NF , Ten Dijke P . Therapeutic targeting of TGF‐β in cancer: hacking a master switch of immune suppression. Clin Sci (Lond). 2021;135(1):35–52. 10.1042/CS20201236 PMC7796313 33399850

[39] Klement JD , Paschall AV , Redd PS , Ibrahim ML , Lu C , Yang D , et al. An osteopontin/CD44 immune checkpoint controls CD8+ T cell activation and tumor immune evasion. J Clin Invest. 2018;128(12):5549–5560. 10.1172/JCI123360 PMC6264631 30395540

[40] Salmon H , Franciszkiewicz K , Damotte D , Dieu‐Nosjean MC , Validire P , Trautmann A , et al. Matrix architecture defines the preferential localization and migration of T cells into the stroma of human lung tumors. J Clin Invest. 2012;122:899–910. 10.1172/JCI45817 PMC3287213 22293174

[41] He J , Xue W , Li Y . Tumor microenvironment‐responsive nanomedicine: monitoring and modulating the tumor microenvironment for precision cancer therapy. Int J Nanomedicine. 2026;21:560983. 10.2147/IJN.S560983 PMC13011976 41884284

[42] Hu Q , Zhu Y , Mei J , Liu Y , Zhou G . Extracellular matrix dynamics in tumor immunoregulation: from tumor microenvironment to immunotherapy. J Hematol Oncol. 2025;18:65. 10.1186/s13045-025-01717-y PMC12178005 40537775

[43] Lv W , Yu L , Wang L , Tang S , Yuan J , Wu Z , et al. Development and evaluation of a predictive model based on multi‐frequency magnetic resonance elastography for high‐risk esophagogastric varices in patients with cirrhotic portal hypertension. Insights Imaging. 2026;17(1):69. 10.1186/s13244-026-02246-z PMC12992754 41838355

[44] Amoiradaki K , Tomczyk M , Wang X , Cruz G , Velasco C , Zentilin L , et al. Molecular and functional MRI enables detection of cardiac fibrosis and evaluation of treatment response after chordin‐like 1 gene therapy in myocardial infarction. Theranostics. 2025;15(17):8706–8718. 10.7150/thno.114459 PMC12439134 40963931

[45] Janas A , Jordan J , Bertalan G , Meyer T , Bukatz J , Sack I , et al. In vivo characterization of brain tumor biomechanics: magnetic resonance elastography in intracranial B16 melanoma and GL261 glioma mouse models. Front Oncol. 2024;11(14):1402578. 10.3389/fonc.2024.1402578 PMC11422132 39324003

[46] Mangarova DB , Reimann C . Elastin‐specific MR probe for visualization and evaluation of an interleukin‐1β targeted therapy for atherosclerosis. Sci Rep. 2024;14(1):20648. 10.1038/s41598-024-71716-5 PMC11375012 39232217

[47] Zhong BY , Fan W , Guan JJ , Peng Z , Jia Z , Jin H , et al. Combination locoregional and systemic therapies in hepatocellular carcinoma. Lancet Gastroenterol Hepatol. 2025;10(4):369–386. 10.1016/S2468-1253(24)00247-4 39993404

[48] Shen S , Dean DC , Yu Z , Duan Z . Role of cyclin‐dependent kinases (CDKs) in hepatocellular carcinoma: therapeutic potential of targeting the CDK signaling pathway. Hepatol Res. 2019;49(10):1097–1108. 10.1111/hepr.13353 31009153

[49] Digiacomo G , Fumarola C , La Monica S , Bonelli M , Cavazzoni A , Galetti M , et al. CDK4/6 inhibitors improve the anti‐tumor efficacy of lenvatinib in hepatocarcinoma cells. Front Oncol. 2022;12:942341. 10.3389/fonc.2022.942341 PMC9354684 35936714

[50] Huang CY , Hsieh FS , Wang CY , Chen LJ , Chang SS , Tsai MH , et al. Palbociclib enhances radiosensitivity of hepatocellular carcinoma and cholangiocarcinoma via inhibiting ataxia telangiectasia–mutated kinase–mediated DNA damage response. Eur J Cancer. 2018;102:10–22. 10.1016/j.ejca.2018.07.010 30103095

[51] Yuan T , Qi Z , Zhang X , Yang K , Xu Y , Cao J . An all‐in‐one theranostic platform for enhanced TACE: self‐developing embolization with dual‐targeted arsenic trioxide delivery. J Nanobiotechnol. 2026;24(1):506. 10.1186/s12951-026-04382-6 PMC13217800 41992318

[52] Dimitriou ID , Clemenza L , Scotter AJ , Chen G , Guerra FM , Rottapel R . Putting out the fire: coordinated suppression of the innate and adaptive immune systems by SOCS1 and SOCS3 proteins. Immunol Rev. 2008;224:265–283. 10.1111/j.1600-065X.2008.00659.x 18759933

[53] Lyu N , Kong Y , Li X , Mu L , Deng H , Chen H , et al. Ablation reboots the response in advanced hepatocellular carcinoma with stable or atypical response during PD‐1 therapy: a proof‐of‐concept study. Front Oncol. 2020;10:580241. 10.3389/fonc.2020.580241 PMC7581675 33163408

[54] Qin S , Chen M , Cheng AL , Kaseb AO , Kudo M , Lee HC , et al. Atezolizumab plus bevacizumab versus active surveillance in patients with resected or ablated high‐risk hepatocellular carcinoma (IMbrave050): a randomised, open‐label, multicenter, phase 3 trial. Lancet. 2023;402(10415):1835–1847. 10.1016/S0140-6736(23)01796-8 37871608

[55] Liang P , Yu J , Wang L , Chen ST , Dong G , Hou QD , et al. A first‐in‐human study to evaluate a personalized neoantigen‐based mRNA‐loaded dendritic cell vaccine, in combination with ablation, in patients with hepatocellular carcinoma. J Clin Oncol. 2024;42(16):e14513.

[56] Pascale F , Pelage JP , Wassef M , Ghegediban SH , Saint‐Maurice JP , de Baere T , et al. Rabbit VX2 liver tumor model: a review of clinical, biology, histology, and tumor microenvironment characteristics. Front Oncol. 2022;12:871829. 10.3389/fonc.2022.871829 PMC9128410 35619923

[57] Primmer CR , Papakostas S , Leder EH , Davis MJ , Ragan MA . Annotated genes and nonannotated genomes: cross‐species use of gene ontology in ecology and evolution research. Mol Ecol. 2013;22(12):3216–3241. 10.1111/mec.12309 23763602

