# Supplementary material for: Extracellular matrix remodeling and immune reprogramming drive residual tumor progression of liver cancer after incomplete microwave ablation
Source: Mol Oncol. 2026 Sep 22:10.1002/1878-0261.70337. Online ahead of print. doi: 10.1002/1878-0261.70337 (PMC13598307; doi:10.1002/1878-0261.70337)
Supplement: Supplementary file 1 [file MOL2-9999-0-s001.docx]

**Supporting Information**

**Supplementary Figure S1. Heatmap of differentially expressed proteins across Day 0 (D0), Day 3 (D3), and Day 4 (D14) groups.**

**Supplementary Table S1. List of upregulated proteins at D3 and D14.**

This table summarizes the 81 proteins that were upregulated at D3 and the 92 proteins with elevated expression at D14 identified by proteomic analysis in conjunction with the transcriptional dataset. The orange-highlighted proteins indicate those commonly present in both the D3 and D14 groups.

**Supplementary Table S2. List of the functional enrichments of protein network for D3 group.**

This table presents the functional enrichments of the protein network from STRING analysis for D3 group. A signal value of > 2 served as the cut-off for pathway inclusion.

**Supplementary Table S3. List of the functional enrichments of protein networks for D14 group.**

This table presents the functional enrichments of the protein networks from STRING analysis for D14 group. A signal value of > 2 served as the cut-off for pathway inclusion.

**Supplementary Table S4. Enriched pathways of upregulated proteins at D3.**

This table displays the functional enrichment analysis of upregulated proteins for D3 using Enrichr. Only pathways with an adjusted P-value of less than 0.05.

**Supplementary Table S5. Enriched pathways of upregulated proteins at D14.**

This table displays the functional enrichment analysis of upregulated proteins for D14 using Enrichr. Only pathways with an adjusted P-value of less than 0.05

**Supplementary Table S6. Fold changes of markers/regulators for macrophages and immune checkpoints.**

This table lists the fold change of various markers or regulators for macrophages and immune checkpoints for D3 and D14 compared with baseline, respectively.

**Supplementary Table S7. Canonical gene sets used for stouffer analysis.**

This table lists the Canonical gene sets utilized for the stouffer analysis for D3 and D14 groups, respectively.

**Supplementary Figure S1. Heatmap of differentially expressed proteins across Day 0 (D0), Day 3 (D3), and Day 4 (D14) .**

Heatmap depicting the differentially expressed proteins. The color intensity represents the relative protein abundance, with red indicating high expression and blue indicating low expression. The heatmap includes the D0 (n = 5), D3 (n = 7), and D14 (n = 5) groups. D and S denote Day and Sample, respectively (e.g., D0 S1 indicates Sample 1 collected on Day 0).

**Supplementary Table S1. List of upregulated proteins at D3 and D14.**

| **D3** | | | **D14** | | | **sharing proteins** |
| --- | --- | --- | --- | --- | --- | --- |
| ADAM19 | GP9 | S100A9 | C1QA | MMP1 | TRIM24 | ADAM8 |
| ADAM8 | ICAM3 | SAA2 | C1QB | MMP2 | ANXA8 | ANXA1 |
| ALG6 | IL1B | SAA3 | C1QC | MMP9 | ABI3BP | CLU |
| C4BPA | ITGA2 | SAA4 | CD14 | MMP12 | PDLIM4 | FN1 |
| C5 | ITGA2B | SELP | CD68 | ADAM8 | NR4A2 | IGF1R |
| C6 | ITGAD | SERPINA3 | CD300LF | TIMP1 | PPP6R2 | IL1RN |
| C8B | ITGAM | SERPINF2 | GPNMB | TIMP3 | SLC2A1 | ITGA2 |
| C8G | ITGB2 | SERPING1 | CHI3L1 | HIF1A | ARFGEF3 | LGALS3 |
| C9 | ITGB3 | ST6GAL1 | HMOX1 | JAG1 | CUL9 | LOXL2 |
| CCL14 | ITGB6 | TFPI | LGALS3 | ANGPTL2 | RAB25 | MMP1 |
| CDH3 | KLKB1 | TGFB2 | LGALS3BP | EPHB3 | BACH1 | MMP9 |
| CHST2 | KNG1 | THBS1 | TGFB2 | FN1 | TP63 | P3H2 |
| CLEC16A | KRT23 | THBS2 | TGFBI | THBS2 | TRIM29 | PROS1 |
| CLU | KRT3 | TIMP3 | TGFBR1 | THBS3 | GINS3 | SELP |
| CRP | LBP | UGT1 | ANXA1 | THBS4 | TSEN2 | TGFB2 |
| CSGALNACT2 | LOXL2 | UGT2B13 | VCAN | LOX | FAM83G | THBS2 |
| CXCL8 | LOXL4 | UGT2C1 | CLU | LOXL2 | HAUS2 | TIMP3 |
| CXCR1 | MAN1A2 | UGT8 | FMOD | SRPX | HAUS3 |  |
| DSC3 | MANBA | Ugt1a4 | IL1RN | SRPX2 | CAMP |  |
| DSCC1 | MMP1 | S100A9 | IKZF3 | BARD1 | ITGA2 |  |
| EXTL1 | MMP10 |  | PDCD11 | EMP1 | ANXA1 |  |
| F10 | MMP3 |  | NDRG1 | CEP55 | SELP |  |
| F11 | MMP8 |  | CDK1 | JADE3 | LGALS3 |  |
| F13B | MMP9 |  | CDK2 | RASSF6 | ITGA6 |  |
| F5 | P3H2 |  | CDK4 | P3H2 | IL1RN |  |
| F9 | POMGNT1 |  | CDK6 | BMP1 | PROS1 |  |
| FGB | PROC |  | CDKN2B | IGF1R | IGF1R |  |
| FGG | PROS1 |  | CD44 | IGF2BP1 | PLD2 |  |
| FN1 | S100A12 |  | BRAF | IGF2BP3 | BSG |  |
| FPR1 | S100A14 |  | PIK3CD | CDK12 | SLC7A1 |  |
| FUT8 | S100A8 |  | HTRA1 | KLF5 |  |  |

| **Mollecular Function(Gene ontology)**  **Supplementary Table S2. List of the functional enrichments of protein network for D3 group.**  T  05 are included. | | | | | | | |
| --- | --- | --- | --- | --- | --- | --- | --- |
| **term ID** | **term description** | **observed  gene count** | **background  gene count** | **strength** | **signal** | **FDR** | **matching proteins in the network** |
| GO:0007596 | Blood coagulation | 15 | 87 | 1.66 | 3.88 | 1.44e-16 | SERPING1,F13B,F9,F11,TFPI,FGB,KLKB1,GP9,PROS1,F10,F5,FGG,KNG1,ITGB3,PROC |
| GO:0033627 | Cell adhesion mediated by integrin | 7 | 37 | 1.7 | 1.99 | 1.09e-07 | ITGA2B,ITGAM,ITGB6,ITGA2,ITGAD,ITGB3,ITGB2 |
| GO:0042060 | Wound healing | 19 | 213 | 1.38 | 3.17 | 1.44e-16 | SERPING1,F13B,F9,TGFB2,F11,TFPI,FGB,KLKB1,GP9,PROS1,F10,F5,ITGB6,ITGA2,FGG,KNG1,ITGB3,PROC,CDH3 |
| GO:0007160 | Cell-matrix adhesion | 9 | 97 | 1.39 | 1.8 | 9.20e-08 | ITGA2B,FGB,ITGAM,ITGB6,ITGA2,ITGAD,FGG,ITGB3,ITGB2 |
| GO:0098609 | Cell-cell adhesion | 17 | 374 | 1.08 | 1.87 | 4.94e-11 | ITGA2B,TGFB2,S100A9,S100A8,FGB,DSC3,SELP,ICAM3,IL1B,ITGAM,ITGA2,ITGAD,FGG,ITGB3,ITGB2,CDH3,ADAM8 |
| GO:0006954 | Inflammatory response | 22 | 368 | 1.2 | 2.68 | 1.44e-16 | SERPINF2,SAA3,THBS1,CRP,C5,CLU,S100A9,S100A8,FN1,KLKB1,SELP,CHST2,S100A12,CCL14,CXCL8,IL1B,SAA2,ITGB6,KNG1,ITGB2,ADAM8,LBP |
| GO:0030574 | Collagen catabolic process | 5 | 30 | 1.65 | 1.31 | 3.41e-05 | MMP8,MMP3,MMP10,MMP9,MMP1 |
| GO:0042730 | Fibrinolysis | 5 | 11 | 2.08 | 1.85 | 9.02e-07 | SERPING1,FGB,KLKB1,PROS1,FGG |
| GO:0006953 | Acute-phase response | 7 | 24 | 1.89 | 2.35 | 9.93e-09 | SERPINF2,SAA3,CRP,FN1,IL1B,SAA2,LBP |
| GO:0032963 | Collagen metabolic process | 6 | 49 | 1.52 | 1.38 | 1.33e-05 | MMP8,P3H2,MMP3,MMP10,MMP9,MMP1 |
| **Cellular Component(Gene ontology)** | | | | | | | |
| **term ID** | **term description** | **observed  gene count** | **background  gene count** | **strength** | **signal** | **FDR** | **matching proteins in the network** |
| GO:0031091 | Platelet alpha granule | 6 | 19 | 1.93 | 2.09 | 9.99e-08 | THBS1,FGB,SELP,THBS2,F5,FGG |
| GO:0042060 | Wound healing | 19 | 213 | 1.38 | 3.17 | 1.44e-16 | SERPING1,F13B,F9,TGFB2,F11,TFPI,FGB,KLKB1,GP9,PROS1,F10,F5,ITGB6,ITGA2,FGG,KNG1,ITGB3,PROC,CDH3 |
| GO:0031012 | Extracellular matrix | 15 | 362 | 1.04 | 1.6 | 4.28e-09 | SERPINF2,MMP8,THBS1,TGFB2,TIMP3,P3H2,LOXL2,MMP3,MMP10,THBS2,MMP9,FGG,MMP1,LOXL4,ADAM19 |
| GO:0008305 | Integrin complex | 7 | 28 | 1.83 | 2.27 | 1.52e-08 | ITGA2B,ITGAM,ITGB6,ITGA2,ITGAD,ITGB3,ITGB2 |
| **Reactome Pathways** | | | | | | | |
| **term ID** | **term description** | **observed  gene count** | **background  gene count** | **strength** | **signal** | **FDR** | **matching proteins in the network** |
| MAP-3000178 | ECM proteoglycans | 5 | 52 | 1.41 | 1.08 | 0.00015 | ITGA2B,FN1,ITGB6,ITGA2,ITGB3 |
| GO:0042060 | Wound healing | 19 | 213 | 1.38 | 3.17 | 1.44e-16 | SERPING1,F13B,F9,TGFB2,F11,TFPI,FGB,KLKB1,GP9,PROS1,F10,F5,ITGB6,ITGA2,FGG,KNG1,ITGB3,PROC,CDH3 |
| MAP-354194 | GRB2:SOS provides linkage to MAPK signaling for Integrins | 5 | 19 | 1.85 | 1.65 | 3.07e-06 | ITGA2B,FN1,FGB,FGG,ITGB3 |
| MAP-372708 | p130Cas linkage to MAPK signaling for integrins | 5 | 21 | 1.8 | 1.6 | 4.13e-06 | ITGA2B,FN1,FGB,FGG,ITGB3 |
| MAP-166658 | Complement cascade | 8 | 113 | 1.28 | 1.42 | 2.32e-06 | SERPING1,CRP,C5,CLU,C9,C4BPA,C8B,C6 |
| MAP-1566948 | Elastic fibre formation | 6 | 53 | 1.48 | 1.42 | 7.96e-06 | TGFB2,FN1,LOXL2,ITGB6,ITGB3,LOXL4 |

| **Biological Process (Gene ontology)**  **Supplementary Table S3. List of the functional enrichments of protein network for D14 group.**  T  05 are included. | | | | | | | |
| --- | --- | --- | --- | --- | --- | --- | --- |
| **term ID** | **term description** | **observed  gene count** | **background  gene count** | **strength** | **signal** | **FDR** | **matching proteins in the network** |
| GO:0042127 | Regulation of cell population proliferation | 31 | 1278 | 0.75 | 1.34 | 9.64e-12 | IKZF3,CDK1,JAG1,TGFB2,ABI3BP,CLU,SRPX,MMP12,THBS4,HMOX1,TIMP1,P3H2,CDKN2B,CDK4,HTRA1,IGF1R,ANXA1,PIK3CD,MMP9,ITGA2,BRAF,TRIM24,CDK2,TGFBR1,CDK6,GPNMB,HIF1A,NDRG1,SLC7A1,RAB25,TP63 |
| GO:0006954 | Inflammatory response | 14 | 368 | 0.95 | 1.23 | 7.10e-07 | CD14,CLU,FN1,CD68,HMOX1,TIMP1,SELP,ANXA1,CHI3L1,IL1RN,PIK3CD,C1QA,HIF1A,ADAM8 |
| GO:0030335 | Positive regulation of cell migration | 15 | 425 | 0.92 | 1.21 | 5.94e-07 | ITGA6,TGFB2,THBS4,HMOX1,SRPX2,IGF1R,SELP,ANXA1,PIK3CD,MMP9,ITGA2,TGFBR1,GPNMB,HIF1A,ADAM8 |
| GO:0071492 | Cellular response | 4 | 11 | 1.93 | 1.21 | 9.20e-05 | TIMP1,MMP9,MMP2,MMP1 |
| GO:0008284 | Positive regulation of cell population proliferation | 20 | 707 | 0.82 | 1.2 | 6.16e-08 | CDK1,TGFB2,MMP12,THBS4,HMOX1,TIMP1,CDK4,HTRA1,IGF1R,ANXA1,PIK3CD,MMP9,ITGA2,CDK2,TGFBR1,CDK6,HIF1A,SLC7A1,RAB25,TP63 |
| GO:0010942 | Positive regulation of cell death | 15 | 458 | 0.88 | 1.15 | 8.42e-07 | ITGA6,BARD1,TGFB2,CLU,SRPX,TIMP3,HMOX1,HTRA1,ANXA1,PIK3CD,MMP9,C1QA,TGFBR1,ADAM8,TP63 |
| GO:0050678 | Regulation of epithelial cell proliferation | 12 | 299 | 0.97 | 1.15 | 3.65e-06 | JAG1,TGFB2,MMP12,THBS4,HMOX1,CDKN2B,HTRA1,PIK3CD,TRIM24,TGFBR1,CDK6,TP63 |
| GO:0043065 | Positive regulation of apoptotic process | 14 | 407 | 0.91 | 1.14 | 1.56e-06 | ITGA6,BARD1,TGFB2,CLU,SRPX,TIMP3,HMOX1,HTRA1,ANXA1,PIK3CD,MMP9,TGFBR1,ADAM8,TP63 |
| GO:0022407 | Regulation of cell-cell adhesion | 18 | 355 | 1.13 | 2.15 | 7.72e-13 | PPM1F,DOCK8,IDO1,CD74,PYCARD,PNP,ZAP70,ADA,PTPRC,NCKAP1L,JAK2,SYK,CORO1A,PODXL,ARG1,PRKCD,TGFBR2,RDX |
| **Molecular Function(Gene ontology)** | | | | | | | |
| **term ID** | **term description** | **observed  gene count** | **background  gene count** | **strength** | **signal** | **FDR** | **matching proteins in the network** |
| GO:0030332 | Cyclin binding | 6 | 37 | 1.58 | 1.16 | 8.50e-05 | CDK12,CDK1,CDK4,HAUS3,CDK2,CDK6 |
| GO:0008353 | RNA polymerase II CTD heptapeptide repeat kinase activity | 4 | 10 | 1.97 | 1.02 | 0.00043 | CDK12,CDK1,CDK4,CDK6 |
| GO:0004693 | Cyclin-dependent proteinserine/threonine kinase activity | 5 | 28 | 1.62 | 0.98 | 0.00043 | CDK12,CDK1,CDK4,CDK2,CDK6 |
| GO:0005539 | Glycosaminoglycan binding | 9 | 206 | 1.01 | 0.83 | 0.00043 | VCAN,ABI3BP,THBS4,THBS3,FN1,SELP,THBS2,GPNMB,CD44 |
| GO:0008201 | Heparin binding | 7 | 141 | 1.07 | 0.75 | 0.0012 | ABI3BP,THBS4,THBS3,FN1,SELP,THBS2,GPNMB |
| GO:0019838 | Growth factor binding | 6 | 103 | 1.13 | 0.72 | 0.0021 | ITGA6,SRPX2,HTRA1,IGF1R,IL1RN,TGFBR1 |
| GO:0005518 | Collagen binding | 5 | 65 | 1.26 | 0.7 | 0.0032 | ABI3BP,MMP12,ITGA2,TGFBI,LOX |
| **Reactome Pathways** | | | | | | | |
| **term ID** | **term description** | **observed  gene count** | **background  gene count** | **strength** | **signal** | **FDR** | **matching proteins in the network** |
| MAP-1474244 | Extracellular matrix organization | 17 | 338 | 1.07 | 1.81 | 2.29e-10 | ITGA6,VCAN,TGFB2,MMP12,FN1,TIMP1,P3H2,HTRA1,LOXL2,MMP9,BMP1,ITGA2,MMP2,MMP1,LOX,ADAM8,CD44 |
| MAP-1474228 | Degradation of the extracellular matrix | 9 | 142 | 1.17 | 1.19 | 1.38e-05 | MMP12,FN1,TIMP1,HTRA1,MMP9,MMP2,MMP1,ADAM8,CD44 |

**Supplementary Table 2. Enriched pathways of upregulated proteins at D 3.**

This table displays the functional enrichment analysis of upregulated proteins for D3 using Enrichr. Only pathways with an adjusted P-value of less than 0.05.

05 are included.

| **term**  **Supplementary Table S4. Enriched pathways of upregulated proteins at D3**  T  05 are included. | **adjusted P-value** | **Odds Ratio** | **combined score** | **category** |
| --- | --- | --- | --- | --- |
| Dengue 2 Interactions With Complement And Coagulation Cascades WP3896 | 7.22E-24 | 113.78 | 6,545.72 | Acute injury response |
| Formation of Fibrin Clot (Clotting Cascade) | 1.77E-22 | 166.28 | 9,138.07 | Acute injury response |
| Hemostasis | 8.81E-19 | 14.97 | 689.14 | Acute injury response |
| Platelet Degranulation | 4.64E-18 | 43.53 | 1,919.19 | Acute injury response |
| Response to Elevated Platelet Cytosolic Ca2+ | 7.01E-18 | 41.66 | 1,810.21 | Acute injury response |
| Blood Clotting Cascade WP272 | 4.61E-15 | 191.40 | 7,075.45 | Acute injury response |
| Intrinsic Pathway of Fibrin Clot Formation | 6.26E-15 | 177.72 | 6,482.08 | Acute injury response |
| Platelet Activation, Signaling and Aggregation | 1.32E-14 | 21.33 | 758.78 | Acute injury response |
| Common Pathway of Fibrin Clot Formation | 5.26E-11 | 125.52 | 3,367.84 | Acute injury response |
| Diseases of Hemostasis | 1.77E-09 | 122.50 | 2,826.85 | Acute injury response |
| Platelet Aggregation (Plug Formation) | 1.33E-07 | 48.21 | 884.69 | Acute injury response |
| Extrinsic Pathway of Fibrin Clot Formation | 4.40E-06 | 383.02 | 5,464.83 | Acute injury response |
| Burn Wound Healing WP5055 | 5.98E-06 | 23.35 | 336.88 | Acute injury response |
| Fibrin Complement Receptor 3 Signaling WP4136 | 8.57E-06 | 34.42 | 482.35 | Acute injury response |
| COVID 19 Thrombosis And Anticoagulation WP4927 | 1.85E-05 | 191.49 | 2,493.37 | Acute injury response |
| Platelet Mediated Interactions W Vascular And Circulating Cells WP4462 | 2.53E-04 | 54.68 | 551.40 | Acute injury response |
| IL1 And Megakaryocytes In Obesity WP2865 | 6.24E-04 | 36.44 | 328.46 | Acute injury response |
| Spinal Cord Injury WP2431 | 6.24E-04 | 11.43 | 102.96 | Acute injury response |
| Neovascularization Processes WP4331 | 2.94E-02 | 14.38 | 66.46 | Angiogenesis/Vascular |
| Complement System WP2806 | 7.22E-24 | 73.63 | 4,238.93 | Complement system |
| Complement System In Neuronal Development And Plasticity WP5090 | 1.42E-14 | 40.76 | 1,451.70 | Complement system |
| Terminal Pathway of Complement | 2.64E-12 | 796.68 | 23,834.81 | Complement system |
| Complement | 3.19E-10 | 18.25 | 443.67 | Complement system |
| Defects of Contact Activation System (CAS) and Kallikrein Kinin System (KKS) | 6.14E-10 | 159.27 | 3,868.29 | Complement system |
| Complement Cascade | 4.06E-09 | 21.61 | 479.16 | Complement system |
| Regulation of Complement Cascade | 3.67E-08 | 20.80 | 412.81 | Complement system |
| Complement Activation WP545 | 5.32E-07 | 72.74 | 1,258.65 | Complement system |
| Toll Like Receptor 4 (TLR4) Cascade | 1.27E-06 | 15.48 | 243.12 | Complement system |
| Toll-like Receptor Cascades | 4.00E-06 | 13.04 | 188.61 | Complement system |
| Kinin Kallikrein Pathway WP5089 | 1.85E-05 | 191.49 | 2,493.37 | Complement system |
| RAS And Bradykinin Pathways In COVID 19 WP4969 | 4.41E-05 | 41.34 | 500.72 | Complement system |
| Diseases Associated With the TLR Signaling Cascade | 5.83E-05 | 33.33 | 377.95 | Complement system |
| Complement Mediated Inflam Of Pulmonary Alveolus In COV19 WP5148 | 9.75E-05 | 85.09 | 952.72 | Complement system |
| MyD88 MAL(TIRAP) Cascade Initiated on Plasma Membrane | 4.44E-04 | 11.85 | 108.64 | Complement system |
| Toll Like Receptor TLR6 TLR2 Cascade | 4.44E-04 | 11.85 | 108.64 | Complement system |
| Toll Like Receptor 2 (TLR2) Cascade | 4.71E-04 | 11.53 | 104.33 | Complement system |
| Toll Like Receptor TLR1 TLR2 Cascade | 4.71E-04 | 11.53 | 104.33 | Complement system |
| MAPK Family Signaling Cascades | 5.91E-03 | 5.09 | 32.40 | Complement system |
| RAF MAP Kinase Cascade | 1.36E-02 | 4.97 | 26.94 | Complement system |
| Extracellular Matrix Organization | 1.77E-22 | 29.97 | 1,666.14 | ECM Remodeling/Fibrosis |
| Integrin Cell Surface Interactions | 2.81E-14 | 47.28 | 1,639.91 | ECM Remodeling/Fibrosis |
| p130Cas Linkage to MAPK Signaling for Integrins | 3.83E-08 | 130.98 | 2,579.66 | ECM Remodeling/Fibrosis |
| GRB2 SOS Provides Linkage to MAPK Signaling for Integrins | 3.83E-08 | 130.98 | 2,579.66 | ECM Remodeling/Fibrosis |
| Activation of Matrix Metalloproteinases | 4.82E-08 | 58.94 | 1,144.15 | ECM Remodeling/Fibrosis |
| Matrix Metalloproteinases WP129 | 5.44E-08 | 66.32 | 1,328.07 | ECM Remodeling/Fibrosis |
| Elastic Fibre Formation | 2.70E-07 | 41.85 | 736.46 | ECM Remodeling/Fibrosis |
| Integrin Signaling | 6.72E-07 | 59.50 | 978.28 | ECM Remodeling/Fibrosis |
| Degradation of the Extracellular Matrix | 8.55E-07 | 16.43 | 265.01 | ECM Remodeling/Fibrosis |
| Integrin Mediated Cell Adhesion WP185 | 2.27E-06 | 19.95 | 310.54 | ECM Remodeling/Fibrosis |
| ECM Proteoglycans | 4.40E-06 | 22.68 | 323.61 | ECM Remodeling/Fibrosis |
| Focal Adhesion PI3K Akt mTOR Signaling WP3932 | 4.63E-06 | 9.43 | 139.00 | ECM Remodeling/Fibrosis |
| miRNA Targets In ECM And Membrane Receptors WP2911 | 1.58E-05 | 57.43 | 762.09 | ECM Remodeling/Fibrosis |
| Syndecan Interactions | 2.26E-05 | 44.94 | 557.64 | ECM Remodeling/Fibrosis |
| Collagen Degradation | 3.23E-05 | 22.15 | 265.98 | ECM Remodeling/Fibrosis |
| Molecules Associated With Elastic Fibres | 7.17E-05 | 31.30 | 347.94 | ECM Remodeling/Fibrosis |
| Focal Adhesion WP306 | 1.09E-04 | 9.72 | 107.20 | ECM Remodeling/Fibrosis |
| Collagen Formation | 1.50E-04 | 15.35 | 158.75 | ECM Remodeling/Fibrosis |
| Non-integrin membrane-ECM Interactions | 4.23E-04 | 18.76 | 173.58 | ECM Remodeling/Fibrosis |
| Assembly of Collagen Fibrils and Other Multimeric Structures | 4.51E-04 | 18.10 | 165.11 | ECM Remodeling/Fibrosis |
| Osteopontin Signaling WP1434 | 5.03E-03 | 45.82 | 307.11 | ECM Remodeling/Fibrosis |
| Platelet Adhesion to Exposed Collagen | 6.33E-03 | 35.99 | 226.04 | ECM Remodeling/Fibrosis |
| miR 509 3P Alteration Of YAP1 ECM Axis WP3967 | 7.82E-03 | 33.59 | 206.84 | ECM Remodeling/Fibrosis |
| Endochondral Ossification WP474 | 7.82E-03 | 12.73 | 78.29 | ECM Remodeling/Fibrosis |
| Endochondral Ossification With Skeletal Dysplasias WP4808 | 7.82E-03 | 12.73 | 78.29 | ECM Remodeling/Fibrosis |
| Crosslinking of Collagen Fibrils | 7.92E-03 | 31.49 | 190.28 | ECM Remodeling/Fibrosis |
| Turbulent Flow Shear Stress Activates Signaling by PIEZO1 and Integrins in Endothelial Cells | 1.97E-02 | 17.36 | 86.20 | ECM Remodeling/Fibrosis |
| Innate Immune System | 7.82E-13 | 8.38 | 261.69 | Immune Function |
| Immune System | 1.60E-09 | 5.21 | 121.08 | Immune Function |
| Interleukin-4 and Interleukin-13 Signaling | 1.18E-08 | 24.05 | 505.99 | Immune Function |
| Network Map Of SARS CoV 2 Signaling WP5115 | 1.16E-06 | 11.36 | 185.89 | Immune Function |
| Neutrophil Degranulation | 3.78E-06 | 7.26 | 105.62 | Immune Function |
| Signaling by Interleukins | 1.15E-05 | 6.94 | 90.97 | Immune Function |
| Antiviral And Anti-Inflam Effects Of Nrf2 On SARS CoV 2 Pathway WP5113 | 5.59E-05 | 38.27 | 453.05 | Immune Function |
| Diseases of Immune System | 5.83E-05 | 33.33 | 377.95 | Immune Function |
| Interleukin-10 Signaling | 3.09E-03 | 17.78 | 125.55 | Immune Function |
| Nanomaterial Induced Inflammasome Activation WP3890 | 6.58E-02 | 41.49 | 148.32 | Immune Function |
| EPAC1 And PKA Reduction Of Retinal Inflammation WP5440 | 7.42E-02 | 35.56 | 122.44 | Immune Function |
| Extrafollicular And Follicular B Cell Activation By SARS CoV 2 WP5218 | 8.24E-02 | 6.98 | 23.15 | Immune Function |
| Hippo Merlin Signaling Dysregulation WP4541 | 5.32E-07 | 19.38 | 335.97 | Signal Transduction |
| KRAS Signaling Up | 6.73E-07 | 12.91 | 206.07 | Signal Transduction |
| Signaling by High-Kinase Activity BRAF Mutants | 2.67E-06 | 42.21 | 630.11 | Signal Transduction |
| Photodynamic Therapy Induced NF kB Survival Signaling WP3617 | 3.49E-06 | 43.62 | 657.53 | Signal Transduction |
| MAP2K and MAPK Activation | 4.21E-06 | 37.38 | 537.64 | Signal Transduction |
| Signaling by RAF1 Mutants | 4.40E-06 | 36.34 | 518.07 | Signal Transduction |
| COVID 19 Adverse Outcome Pathway WP4891 | 6.61E-03 | 38.77 | 248.51 | Signal Transduction |
| Canonical And Non Canonical TGF B Signaling WP3874 | 7.82E-03 | 33.59 | 206.84 | Signal Transduction |
| Interactions Between LOXL4 And Oxidative Stress Pathway WP3670 | 8.56E-03 | 31.49 | 190.28 | Signal Transduction |
| LTF Danger Signal Response Pathway WP4478 | 9.38E-03 | 29.64 | 175.86 | Signal Transduction |
| KRAS Signaling Dn | 9.31E-02 | 3.85 | 11.72 | Signal Transduction |
| IL-6/JAK/STAT3 Signaling | 9.31E-02 | 5.91 | 17.87 | Signal Transduction |
| Orexin Receptor Pathway WP5094 | 1.06E-01 | 5.84 | 17.54 | Signal Transduction |
| Epithelial Mesenchymal Transition | 6.20E-08 | 14.62 | 272.50 | Tumorigenesis |
| Immune Infiltration In Pancreatic Cancer WP5285 | 1.20E-04 | 29.51 | 321.73 | Tumorigenesis |
| Hepatitis C And Hepatocellular Carcinoma WP3646 | 4.31E-03 | 16.62 | 114.27 | Tumorigenesis |
| Small Cell Lung Cancer WP4658 | 2.15E-02 | 8.29 | 41.39 | Tumorigenesis |
| Neural Crest Cell Migration In Cancer WP4565 | 3.71E-02 | 12.27 | 53.17 | Tumorigenesis |
| Cells And Molecules Involved In Acute Inflammatory Response WP4493 | 1.52E-09 | 144.79 | 3,453.88 | Other-Uncategorized |
| Vitamin B12 Metabolism WP1533 | 5.44E-08 | 40.00 | 799.47 | Other-Uncategorized |
| Overview Of Nanoparticle Effects WP3287 | 2.48E-07 | 93.54 | 1,716.80 | Other-Uncategorized |
| Folate Metabolism WP176 | 2.53E-07 | 30.30 | 552.32 | Other-Uncategorized |
| Regulation of TLR by Endogenous Ligand | 2.71E-07 | 77.02 | 1,351.39 | Other-Uncategorized |
| Defective Factor IX Causes Hemophilia B | 3.22E-07 | 206.90 | 3,577.34 | Other-Uncategorized |
| Gamma-carboxylated Protein Precursors Transport From the Endoplasmic Reticulum to Golgi Apparatus | 3.22E-07 | 206.90 | 3,577.34 | Other-Uncategorized |
| Gamma-carboxylation of Protein Precursors | 4.96E-07 | 172.41 | 2,893.43 | Other-Uncategorized |
| Removal of Aminoterminal Propeptides From Gamma-Carboxylated Proteins | 4.96E-07 | 172.41 | 2,893.43 | Other-Uncategorized |
| Gamma-carboxylation, Transport, and Amino-Terminal Cleavage of Proteins | 7.23E-07 | 147.77 | 2,413.62 | Other-Uncategorized |
| Selenium Micronutrient Network WP15 | 9.16E-07 | 23.76 | 396.26 | Other-Uncategorized |
| Vitamin K Metabolism And Activation Of Dependent Proteins WP5186 | 2.27E-06 | 114.92 | 1,788.92 | Other-Uncategorized |
| MyD88 Deficiency (TLR2 4) | 6.16E-06 | 68.93 | 957.79 | Other-Uncategorized |
| IRAK4 Deficiency (TLR2 4) | 6.80E-06 | 64.62 | 883.66 | Other-Uncategorized |
| Defective F9 Activation | 7.33E-06 | 255.33 | 3,466.80 | Other-Uncategorized |
| Chronic Hyperglycemia Impairment Of Neuron Function WP5283 | 1.15E-05 | 31.90 | 436.02 | Other-Uncategorized |
| Allograft Rejection WP2328 | 1.58E-05 | 18.89 | 250.56 | Other-Uncategorized |
| Regulation of IGF Transport and Uptake by Insulin-like Growth Factor Binding Proteins (IGFBPs) | 5.69E-05 | 13.42 | 153.08 | Other-Uncategorized |
| Overview Of Proinflammatory And Profibrotic Mediators WP5095 | 9.75E-05 | 12.88 | 143.91 | Other-Uncategorized |
| Post-translational Protein Phosphorylation | 3.38E-04 | 12.78 | 121.57 | Other-Uncategorized |
| Disease | 3.78E-04 | 2.95 | 27.71 | Other-Uncategorized |
| Pleural Mesothelioma WP5087 | 3.94E-04 | 5.69 | 54.64 | Other-Uncategorized |
| Hematopoietic SC Differentiation WP2849 | 4.11E-04 | 20.24 | 192.83 | Other-Uncategorized |
| N Glycan Biosynthesis WP5153 | 4.49E-04 | 19.47 | 182.79 | Other-Uncategorized |
| Gamma Carboxylation, Hypusinylation, Hydroxylation, and Arylsulfatase Activation | 4.51E-04 | 18.10 | 165.11 | Other-Uncategorized |
| Metal Sequestration by Antimicrobial Proteins | 9.47E-04 | 126.04 | 1,050.32 | Other-Uncategorized |
| Arrhythmogenic Right Ventricular Cardiomyopathy WP2118 | 1.13E-03 | 14.73 | 123.30 | Other-Uncategorized |
| Defective Factor VIII Causes Hemophilia A | 1.27E-03 | 100.83 | 806.56 | Other-Uncategorized |
| Post-translational Protein Modification | 2.48E-03 | 2.91 | 21.25 | Other-Uncategorized |
| Serotonin Transporter Activity WP1455 | 3.95E-03 | 56.01 | 394.66 | Other-Uncategorized |
| Mechanoreg And Pathology Of YAP TAZ Hippo And Non Hippo Mechs WP4534 | 4.06E-03 | 17.37 | 121.60 | Other-Uncategorized |
| O-linked Glycosylation | 4.18E-03 | 9.35 | 63.01 | Other-Uncategorized |
| Xenobiotic Metabolism | 5.05E-03 | 6.65 | 44.13 | Other-Uncategorized |
| Inflammatory Response | 5.05E-03 | 6.65 | 44.13 | Other-Uncategorized |
| Lung Fibrosis WP3624 | 7.82E-03 | 12.73 | 78.29 | Other-Uncategorized |
| Diseases of Glycosylation | 9.66E-03 | 7.24 | 42.19 | Other-Uncategorized |
| Diseases Associated With O-glycosylation of Proteins | 9.75E-03 | 11.23 | 65.22 | Other-Uncategorized |
| RHO GTPases Activate NADPH Oxidases | 1.32E-02 | 22.90 | 125.20 | Other-Uncategorized |
| Diseases of Metabolism | 1.35E-02 | 4.99 | 27.13 | Other-Uncategorized |
| N-glycan Antennae Elongation in the Medial trans-Golgi | 1.48E-02 | 20.99 | 111.43 | Other-Uncategorized |
| Antimicrobial Peptides | 1.96E-02 | 8.29 | 41.39 | Other-Uncategorized |
| Transport to the Golgi and Subsequent Modification | 1.97E-02 | 5.63 | 28.03 | Other-Uncategorized |
| Metabolism of Proteins | 2.23E-02 | 2.14 | 10.34 | Other-Uncategorized |
| Asparagine N-linked Glycosylation | 2.23E-02 | 4.29 | 20.67 | Other-Uncategorized |
| Allograft Rejection | 2.27E-02 | 5.23 | 24.70 | Other-Uncategorized |
| Apical Junction | 2.27E-02 | 5.23 | 24.70 | Other-Uncategorized |
| Monoamine Transport WP727 | 2.32E-02 | 16.78 | 82.29 | Other-Uncategorized |
| Defective B3GALTL Causes PpS | 2.78E-02 | 13.98 | 63.89 | Other-Uncategorized |
| Maturation of Spike Protein 9694548 | 2.78E-02 | 13.98 | 63.89 | Other-Uncategorized |
| O-glycosylation of TSR Domain-Containing Proteins | 2.89E-02 | 13.60 | 61.48 | Other-Uncategorized |
| MET Promotes Cell Motility | 3.12E-02 | 12.90 | 57.08 | Other-Uncategorized |
| Ebola Virus Infection In Host WP4217 | 4.27E-02 | 6.04 | 25.17 | Other-Uncategorized |
| 2Q37 Copy Number Variation Syndrome WP5224 | 4.85E-02 | 5.72 | 23.04 | Other-Uncategorized |
| Male Infertility WP4673 | 5.45E-02 | 5.36 | 20.67 | Other-Uncategorized |
| Hepatitis B Infection WP4666 | 5.91E-02 | 5.14 | 19.28 | Other-Uncategorized |
| Arylamine Metabolism WP694 | 5.92E-02 | 49.78 | 185.56 | Other-Uncategorized |
| Nicotine Metabolism In Liver Cells WP1600 | 5.92E-02 | 49.78 | 185.56 | Other-Uncategorized |
| Acquired Partial Lipodystrophy Barraquer Simons Syndrome WP5104 | 8.75E-02 | 27.65 | 89.16 | Other-Uncategorized |
| Steroid Biosynthesis WP496 | 8.75E-02 | 27.65 | 89.16 | Other-Uncategorized |
| Interferon Gamma Response | 9.31E-02 | 3.85 | 11.72 | Other-Uncategorized |
| Acute Viral Myocarditis WP4298 | 1.01E-01 | 6.05 | 18.55 | Other-Uncategorized |
| Biomarkers For Urea Cycle Disorders WP4583 | 1.02E-01 | 22.62 | 68.91 | Other-Uncategorized |

| **term**  **Supplementary Table S5. Enriched pathways of upregulated proteins at D14.**  T  05 are included. | **adjusted P-value** | **odds ratio** | **combined score** | **category** |
| --- | --- | --- | --- | --- |
| Dengue 2 Interactions With Complement And Coagulation Cascades WP3896 | 7.22E-24 | 113.78 | 6,545.72 | Acute injury response |
| Platelet Degranulation | 2.00E-05 | 16.7 | 271.67 | Acute injury response |
| Response to Elevated Platelet Cytosolic Ca2+ | 2.00E-05 | 16.03 | 255.97 | Acute injury response |
| Dengue 2 Complement Coagulation WP3896 | 1.22E-04 | 22.42 | 270.5 | Acute injury response |
| Platelet Mediated Interactions W Vascular And Circulating Cells WP4462 | 1.24E-02 | 31.21 | 187.79 | Acute injury response |
| Burn Wound Healing WP5055 | 9.59E-02 | 6.48 | 20.67 | Acute injury response |
| Cell Surface Interactions at Vascular Wall | 7.95E-03 | 6.42 | 48.34 | Angiogenesis/Vascular |
| Neovascularization Processes WP4331 | 3.07E-04 | 29.03 | 314.46 | Angiogenesis/Vascular |
| VEGFA VEGFR2 Signaling WP3888 | 4.08E-03 | 4.68 | 34.96 | Angiogenesis/Vascular |
| Angiogenesis WP1539 | 2.02E-02 | 21.27 | 113.36 | Angiogenesis/Vascular |
| miRNA Targeting Cancer Growth And Vascularization Glioblastoma WP3593 | 7.58E-02 | 38.58 | 135.2 | Angiogenesis/Vascular |
| Angiogenesis | 3.00E-03 | 21.52 | 163.04 | Angiogenesis/Vascular |
| Regulation of Complement Cascade | 4.79E-03 | 9.81 | 81.73 | Complement system |
| Complement Cascade | 6.28E-03 | 9 | 71.54 | Complement system |
| Complement In Neuronal Development WP5090 | 1.35E-04 | 14.68 | 174.51 | Complement system |
| Complement | 1.63E-03 | 7.53 | 62.78 | Complement system |
| Extracellular Matrix Organization | 1.59E-09 | 14.35 | 380.31 | ECM Remodeling/Fibrosis |
| Degradation of the Extracellular Matrix | 3.35E-04 | 13.01 | 167.37 | ECM Remodeling/Fibrosis |
| ECM Proteoglycans | 1.15E-03 | 17.04 | 184.29 | ECM Remodeling/Fibrosis |
| Integrin Cell Surface Interactions | 1.67E-03 | 15.12 | 155.27 | ECM Remodeling/Fibrosis |
| Elastic Fibre Formation | 1.67E-03 | 23.94 | 242.65 | ECM Remodeling/Fibrosis |
| Collagen Formation | 1.67E-03 | 14.22 | 142.18 | ECM Remodeling/Fibrosis |
| Crosslinking of Collagen Fibrils | 1.88E-03 | 47.38 | 459.09 | ECM Remodeling/Fibrosis |
| Integrin Mediated Cell Adhesion WP185 | 3.27E-02 | 7.22 | 33.44 | ECM Remodeling/Fibrosis |
| Immune System | 8.17E-04 | 3.19 | 36.65 | Immune Function |
| Innate Immune System | 3.07E-03 | 3.45 | 31.17 | Immune Function |
| Interleukin-4 and Interleukin-13 Signaling | 3.17E-03 | 11.29 | 101.07 | Immune Function |
| Neutrophil Degranulation | 1.57E-02 | 4.19 | 28.44 | Immune Function |
| Cytokine Signaling in Immune System | 2.42E-02 | 3.25 | 20.23 | Immune Function |
| Signaling by Interleukins | 3.53E-02 | 3.83 | 21.59 | Immune Function |
| Microglia Pathogen Phagocytosis WP3937 | 4.00E-04 | 26.61 | 279.84 | Immune Function |
| Network Map Of SARS CoV 2 Signaling WP5115 | 5.43E-03 | 5.87 | 41.55 | Immune Function |
| Macrophage Stimulating Protein MSP Signaling WP5353 | 7.80E-03 | 9.18 | 61.22 | Immune Function |
| Neural Crest Cell Migration During Development WP4564 | 4.03E-02 | 12.31 | 53.34 | Immune Function |
| TYROBP Causal Network In Microglia WP3945 | 7.58E-02 | 7.79 | 27.33 | Immune Function |
| Regulation of HMOX1 Expression | 3.99E-03 | 156.16 | 1341.8 | Immune Function |
| T Cell Modulation In Pancreatic Cancer WP5078 | 6.08E-04 | 22.8 | 227.02 | Immune Function |
| Macrophage Markers WP4146 | 4.53E-03 | 66.91 | 489.99 | Immune Function |
| Interferon Gamma Response | 2.21E-02 | 4.85 | 21.72 | Immune Function |
| Inflammatory Response | 2.21E-02 | 4.85 | 21.72 | Immune Function |
| Signaling by Non-Receptor Tyrosine Kinases | 2.61E-03 | 18.77 | 173.6 | Signal Transduction |
| Signaling by PTK6 | 2.61E-03 | 18.77 | 173.6 | Signal Transduction |
| TGFBR3 Regulates TGF-beta Signaling | 7.82E-03 | 78.07 | 591.08 | Signal Transduction |
| Signaling by TGFBR3 | 1.72E-02 | 15.42 | 102.89 | Signal Transduction |
| Signaling by PDGF | 2.42E-02 | 12.89 | 79.73 | Signal Transduction |
| Integrated Cancer Pathway WP1971 | 2.08E-06 | 38.74 | 664.95 | Signal Transduction |
| Growth Factors In Beta Cell Proliferation WP5385 | 3.58E-06 | 57.76 | 940.44 | Signal Transduction |
| PPAR Alpha Pathway WP2878 | 2.23E-02 | 19.5 | 100.82 | Signal Transduction |
| EGFR Tyrosine Kinase Inhibitor Resistance WP4806 | 2.28E-02 | 8.74 | 44.92 | Signal Transduction |
| Clear Cell Renal Cell Carcinoma Pathways WP4018 | 2.31E-02 | 8.64 | 44.08 | Signal Transduction |
| CAMKK2 Pathway WP4874 | 3.94E-02 | 6.61 | 29.09 | Signal Transduction |
| ATM Signaling WP2516 | 4.03E-02 | 12.31 | 53.34 | Signal Transduction |
| MAPK Signaling WP382 | 6.70E-02 | 3.92 | 14.87 | Signal Transduction |
| NRF2 Pathway WP2884 | 6.91E-02 | 5.12 | 19.16 | Signal Transduction |
| Overlap Between Signal Transduction Pathways LMNA Laminopathies WP4879 | 6.92E-02 | 8.82 | 32.91 | Signal Transduction |
| MET In Type 1 Papillary Renal Cell Carcinoma WP4205 | 7.38E-02 | 8.2 | 29.52 | Signal Transduction |
| Insulin Signaling WP481 | 8.03E-02 | 4.49 | 15.37 | Signal Transduction |
| AGE RAGE Pathway WP2324 | 8.17E-02 | 7.3 | 24.78 | Signal Transduction |
| Ras Signaling WP4223 | 1.05E-01 | 3.89 | 11.96 | Signal Transduction |
| IL-2/STAT5 Signaling | 2.66E-04 | 8.99 | 94.92 | Signal Transduction |
| PI3K/AKT/mTOR Signaling | 5.86E-03 | 9.45 | 64.04 | Signal Transduction |
| Signaling by BRAF and RAF1 Fusions | 3.14E-02 | 11.43 | 66.98 | Signal Transduction |
| Signal Transduction | 3.53E-02 | 2.13 | 12.03 | Signal Transduction |
| PI3K Akt Signaling WP4172 | 7.98E-06 | 8.67 | 132.02 | Signal Transduction |
| TGF B Signaling WP3874 | 2.10E-05 | 73.77 | 1039.33 | Signal Transduction |
| Vitamin D Receptor Pathway WP2877 | 2.75E-04 | 9.65 | 106.07 | Signal Transduction |
| Urotensin II Signaling WP5158 | 2.15E-03 | 14.72 | 123.11 | Signal Transduction |
| TGF Beta Signaling WP366 | 2.41E-03 | 9.5 | 77.78 | Signal Transduction |
| Hippo Merlin Signaling Dysregulation WP4541 | 1.00E-02 | 8.22 | 51.67 | Signal Transduction |
| Inflammatory Response Pathway WP453 | 2.64E-02 | 16.71 | 81.72 | Signal Transduction |
| Signal Transduction Through IL1R WP4496 | 3.07E-02 | 15.09 | 71 | Signal Transduction |
| Mammary Gland Development Pathway Involution Stage 4 Of 4 WP2815 | 9.76E-02 | 25.72 | 81.12 | Signal Transduction |
| Apoptosis | 2.21E-09 | 19.07 | 437.66 | Signal Transduction |
| KRAS Signaling Up | 6.75E-03 | 6.17 | 38.93 | Signal Transduction |
| IL-6/JAK/STAT3 Signaling | 1.90E-02 | 8.43 | 42.49 | Signal Transduction |
| G2-M Checkpoint | 6.75E-03 | 6.17 | 38.93 | Tumorigenesis |
| p53 Pathway | 2.21E-02 | 4.85 | 21.72 | Tumorigenesis |
| Cancer Pathways WP5434 | 1.60E-08 | 9.63 | 227.52 | Tumorigenesis |
| Small Cell Lung Cancer WP4658 | 6.08E-07 | 23.08 | 429.54 | Tumorigenesis |
| Epithelial Mesenchymal Transition Colorectal WP4239 | 8.64E-04 | 9.5 | 90.84 | Tumorigenesis |
| Hepatitis C And Hepatocellular Carcinoma WP3646 | 7.80E-03 | 15.42 | 102.89 | Tumorigenesis |
| NRP1 Triggered Signaling In Pancreatic Cancer WP5144 | 9.16E-03 | 14.19 | 91.41 | Tumorigenesis |
| Immune Infiltration In Pancreatic Cancer WP5285 | 3.96E-02 | 12.64 | 55.4 | Tumorigenesis |
| Bladder Cancer WP2828 | 4.03E-02 | 12.31 | 53.34 | Tumorigenesis |
| Neural Crest Cell Migration In Cancer WP4565 | 4.58E-02 | 11.4 | 47.86 | Tumorigenesis |
| Epithelial Mesenchymal Transition | 3.38E-13 | 22.22 | 720.46 | Tumorigenesis |
| Insulin-like Growth Factor-2 mRNA Binding Proteins Bind RNA | 4.52E-04 | 142.2 | 1754.45 | Other-Uncategorized |
| Cyclin D Associated Events in G1 | 1.67E-03 | 22.27 | 219.83 | Other-Uncategorized |
| G1 Phase | 1.67E-03 | 22.27 | 219.83 | Other-Uncategorized |
| Aberrant Regulation of Mitotic G1 S Transition | 1.67E-03 | 50.76 | 501 | Other-Uncategorized |
| Defective Binding of RB1 Mutants to E2F1 | 1.67E-03 | 50.76 | 501 | Other-Uncategorized |
| Drug-mediated Inhibition of CDK4 CDK6 | 3.99E-03 | 156.16 | 1341.8 | Other-Uncategorized |
| PTK6 Regulates Cell Cycle | 5.30E-03 | 117.11 | 959.14 | Other-Uncategorized |
| Mitotic G1 Phase and G1 S Transition | 6.28E-03 | 9 | 71.54 | Other-Uncategorized |
| PTK6 Promotes HIF1A Stabilization | 6.66E-03 | 93.68 | 736.02 | Other-Uncategorized |
| Senescence-Associated Secretory Phenotype | 7.17E-03 | 12.41 | 96.22 | Other-Uncategorized |
| Oncogene Induced Senescence | 7.59E-03 | 22.19 | 170 | Other-Uncategorized |
| Aberrant Regulation of Mitotic Cell Cycle | 7.82E-03 | 21.52 | 163.04 | Other-Uncategorized |
| Diseases of Mitotic Cell Cycle | 8.61E-03 | 20.28 | 150.45 | Other-Uncategorized |
| NFE2L2 Regulating Anti-Oxidant Enzymes | 3.28E-02 | 27.54 | 159.53 | Other-Uncategorized |
| Loss of Nlp From Mitotic Centrosomes | 3.50E-02 | 10.74 | 61.09 | Other-Uncategorized |
| Loss of Proteins Required for Interphase | 3.50E-02 | 10.74 | 61.09 | Other-Uncategorized |
| AURKA Activation by TPX2 | 3.72E-02 | 10.27 | 57.19 | Other-Uncategorized |
| Insertion of Tail-Anchored Proteins | 3.90E-02 | 23.4 | 128.74 | Other-Uncategorized |
| Transcriptional Regulation by TP53 | 4.47E-02 | 4.07 | 21.58 | Other-Uncategorized |
| Pleural Mesothelioma WP5087 | 5.72E-07 | 8.84 | 167.56 | Other-Uncategorized |
| Glioblastoma Signaling WP2261 | 3.31E-06 | 23.14 | 382.2 | Other-Uncategorized |
| Pancreatic Adenocarcinoma WP4263 | 6.09E-05 | 17.7 | 228.45 | Other-Uncategorized |
| Complement And Coagulation WP558 | 1.20E-04 | 22.85 | 277.56 | Other-Uncategorized |
| G1 To S Cell Cycle Control WP45 | 1.59E-04 | 20.52 | 239.23 | Other-Uncategorized |
| Cell Cycle WP179 | 2.43E-04 | 12.86 | 143.8 | Other-Uncategorized |
| Retinoblastoma Gene In Cancer WP2446 | 5.44E-04 | 14.75 | 149.82 | Other-Uncategorized |
| TROP2 Regulatory Signaling WP5300 | 5.82E-04 | 23.36 | 234.62 | Other-Uncategorized |
| Overview Of Nanoparticle Effects WP3287 | 8.64E-04 | 44.41 | 422.88 | Other-Uncategorized |
| Androgen Receptor In Prostate Cancer WP2263 | 1.29E-03 | 11.61 | 105.49 | Other-Uncategorized |
| TSH Signaling WP2032 | 1.29E-03 | 17.72 | 160.24 | Other-Uncategorized |
| Melanoma WP4685 | 2.15E-03 | 14.95 | 125.88 | Other-Uncategorized |
| DNA Damage Response WP707 | 2.15E-03 | 14.72 | 123.11 | Other-Uncategorized |
| miRNA DNA Damage Response WP1530 | 2.15E-03 | 14.72 | 123.11 | Other-Uncategorized |
| Non Small Cell Lung Cancer WP4255 | 2.41E-03 | 14.06 | 115.35 | Other-Uncategorized |
| Head And Neck Squamous Cell Carcinoma WP4674 | 2.43E-03 | 13.86 | 112.94 | Other-Uncategorized |
| Metabolic Pathways Of Fibroblasts WP5312 | 2.87E-03 | 24.49 | 194.18 | Other-Uncategorized |
| Type I Collagen Synthesis WP4786 | 3.06E-03 | 23.67 | 185.51 | Other-Uncategorized |
| Type 2 Papillary Renal Cell Carcinoma WP4241 | 3.26E-03 | 22.91 | 177.48 | Other-Uncategorized |
| Breast Cancer Pathway WP4262 | 4.08E-03 | 8.09 | 60.5 | Other-Uncategorized |
| HIF1A And PPARG Cardiac Hypertropthy WP2456 | 4.53E-03 | 66.91 | 489.99 | Other-Uncategorized |
| Oxidative Damage Response WP3941 | 4.69E-03 | 19.19 | 139.39 | Other-Uncategorized |
| Mammary Gland Development Pathway Puberty Stage 2 Of 4 WP2814 | 8.51E-03 | 42.57 | 279.32 | Other-Uncategorized |
| Senescence Associated Secretory Phenotype SASP WP3391 | 8.85E-03 | 8.76 | 56.94 | Other-Uncategorized |
| Mod Of PI3K Akt mTOR Signaling By Bioactive Sphingolipids WP5192 | 9.16E-03 | 39.02 | 250.12 | Other-Uncategorized |
| H19 Rb E2F1 And CDK Beta Catenin In Colorectal Cancer WP3969 | 9.16E-03 | 39.02 | 250.12 | Other-Uncategorized |
| 11P11 2 Copy Number Variation Syndrome WP5348 | 1.00E-02 | 13.38 | 84.1 | Other-Uncategorized |
| Ebola Virus Infection In Host WP4217 | 1.24E-02 | 7.63 | 45.92 | Other-Uncategorized |
| Nuclear Receptors Meta Pathway WP2882 | 1.24E-02 | 4.72 | 28.28 | Other-Uncategorized |
| Ferroptosis WP4313 | 1.36E-02 | 11.43 | 66.98 | Other-Uncategorized |
| Measles Virus Infection WP4630 | 1.38E-02 | 7.22 | 42.09 | Other-Uncategorized |
| Acute Myeloid Leukemia WP5293 | 1.50E-02 | 10.91 | 62.48 | Other-Uncategorized |
| Hereditary Leiomyomatosis And Renal Cell Carcinoma Pathway WP4206 | 1.53E-02 | 26.01 | 147.99 | Other-Uncategorized |
| Chromosomal And Microsatellite Instability In Colorectal Cancer WP4216 | 1.78E-02 | 10.12 | 55.97 | Other-Uncategorized |
| Regulation Of Actin Cytoskeleton WP51 | 1.81E-02 | 6.52 | 35.74 | Other-Uncategorized |
| Hepatitis B Infection WP4666 | 1.83E-02 | 6.48 | 35.34 | Other-Uncategorized |
| Kynurenine Pathway And Links To Cell Senescence WP5044 | 2.02E-02 | 21.27 | 113.36 | Other-Uncategorized |
| Relationship Between Inflammation COX 2 And EGFR WP4483 | 2.15E-02 | 20.35 | 106.78 | Other-Uncategorized |
| DNA IR Damage And Cellular Response Via ATR WP4016 | 2.15E-02 | 9.08 | 47.57 | Other-Uncategorized |
| Warburg Effect Mod By Deubiquitinating Enzymes And Substrates WP5216 | 2.23E-02 | 19.5 | 100.82 | Other-Uncategorized |
| Intracellular Trafficking Proteins Involved In CMT Neuropathy WP4856 | 2.31E-02 | 18.72 | 95.4 | Other-Uncategorized |
| Nanoparticle Mediated Activation Of Receptor Signaling WP2643 | 2.39E-02 | 18 | 90.44 | Other-Uncategorized |
| Angiotensin II Receptor Type 1 Pathway WP5036 | 2.39E-02 | 18 | 90.44 | Other-Uncategorized |
| Sphingolipid Metabolism In Senescence WP5121 | 2.39E-02 | 18 | 90.44 | Other-Uncategorized |
| Extracellular Vesicle Mediated Signaling In Recipient Cells WP2870 | 2.53E-02 | 17.33 | 85.9 | Other-Uncategorized |
| Allograft Rejection WP2328 | 2.53E-02 | 8.14 | 40.25 | Other-Uncategorized |
| Tumor Suppressor Activity Of SMARCB1 WP4204 | 2.78E-02 | 16.13 | 77.87 | Other-Uncategorized |
| Regucalcin In Proximal Tubule Epithelial Kidney Cells WP4838 | 2.92E-02 | 15.59 | 74.31 | Other-Uncategorized |
| Prader Willi And Angelman Syndrome WP3998 | 3.32E-02 | 7.15 | 32.91 | Other-Uncategorized |
| P38 MAPK Signaling WP400 | 3.32E-02 | 14.17 | 65.08 | Other-Uncategorized |
| Photodynamic Therapy Induced HIF 1 Survival Signaling WP3614 | 3.66E-02 | 13.36 | 59.92 | Other-Uncategorized |
| Adipogenesis WP236 | 5.88E-02 | 5.52 | 21.73 | Other-Uncategorized |
| Neuroinflammation And Glutamatergic Signaling WP5083 | 6.85E-02 | 5.16 | 19.4 | Other-Uncategorized |
| Oncostatin M Signaling WP2374 | 7.05E-02 | 8.65 | 32.01 | Other-Uncategorized |
| Somatroph Axis GH Relationship To Dietary Restriction And Aging WP4186 | 7.05E-02 | 46.3 | 169.29 | Other-Uncategorized |
| GLP 1 From Intestine And Pancreas Role In Glucose Homeostasis WP5452 | 7.05E-02 | 46.3 | 169.29 | Other-Uncategorized |
| Metastatic Brain Tumor WP2249 | 7.05E-02 | 46.3 | 169.29 | Other-Uncategorized |
| lncRNA Mediated Mechanisms of Therapeutic Resistance WP3672 | 7.05E-02 | 46.3 | 169.29 | Other-Uncategorized |
| Pilocytic Astrocytoma WP2253 | 7.05E-02 | 46.3 | 169.29 | Other-Uncategorized |
| 2Q13 Copy Number Variation Syndrome WP5222 | 7.54E-02 | 8.05 | 28.76 | Other-Uncategorized |
| DYRK1A Involvement Regarding Cell Prolif In Brain Development WP5180 | 7.58E-02 | 7.79 | 27.33 | Other-Uncategorized |
| Amino Acid Metabolism In Triple Negative Breast Cancer Cells WP5213 | 7.58E-02 | 38.58 | 135.2 | Other-Uncategorized |
| Hypothetical Craniofacial Development Pathway WP3655 | 7.58E-02 | 38.58 | 135.2 | Other-Uncategorized |
| LDLRAD4 And What We Know About It WP4904 | 7.58E-02 | 38.58 | 135.2 | Other-Uncategorized |
| GLP 1 In Pancreatic Islet Cells WP5447 | 7.58E-02 | 38.58 | 135.2 | Other-Uncategorized |
| Endometrial Cancer WP4155 | 7.63E-02 | 7.66 | 26.66 | Other-Uncategorized |
| Lung Fibrosis WP3624 | 7.63E-02 | 7.66 | 26.66 | Other-Uncategorized |
| Ketogenesis and Ketolysis WP4742 | 8.22E-02 | 33.07 | 111.54 | Other-Uncategorized |
| Glucose Metabolism In Triple Negative Breast Cancer Cells WP5211 | 8.22E-02 | 33.07 | 111.54 | Other-Uncategorized |
| Th17 Cell Differentiation Pathway WP5130 | 8.65E-02 | 6.97 | 23.1 | Other-Uncategorized |
| Urea Cycle and Related Diseases WP4571 | 9.08E-02 | 28.93 | 94.25 | Other-Uncategorized |
| Arrhythmogenic Right Ventricular Cardiomyopathy WP2118 | 9.59E-02 | 6.48 | 20.67 | Other-Uncategorized |
| TCA Cycle In Senescence WP5050 | 9.76E-02 | 25.72 | 81.12 | Other-Uncategorized |
| IDO Metabolic Pathway WP5414 | 1.05E-01 | 23.14 | 70.84 | Other-Uncategorized |
| NAD Metabolism Sirtuins And Aging WP3630 | 1.05E-01 | 23.14 | 70.84 | Other-Uncategorized |
| Coagulation | 6.12E-09 | 20.07 | 432.12 | Other-Uncategorized |
| Hypoxia | 1.63E-03 | 7.53 | 62.78 | Other-Uncategorized |
| Apical Junction | 6.75E-03 | 6.17 | 38.93 | Other-Uncategorized |
| Cholesterol Homeostasis | 1.41E-02 | 9.98 | 54.8 | Other-Uncategorized |
| UV Response Up | 1.58E-02 | 6.18 | 32.74 | Other-Uncategorized |
| Myogenesis | 2.21E-02 | 4.85 | 21.72 | Other-Uncategorized |
| E2F Targets | 2.21E-02 | 4.85 | 21.72 | Other-Uncategorized |
| Xenobiotic Metabolism | 2.21E-02 | 4.85 | 21.72 | Other-Uncategorized |
| Glycolysis | 2.21E-02 | 4.85 | 21.72 | Other-Uncategorized |
| UV Response Dn | 4.65E-02 | 5.01 | 18.48 | Other-Uncategorized |

| **Category** | **Markers/Regulators** | **D3 VS D0** | **D14 VS D0** | **Change (D14 VS D3)** |
| --- | --- | --- | --- | --- |
| Macrophages  transition and polarization | CD200 | 0.486 | 1.14 | +134.57% |
|  | CD68 | 0.938 | 1.462 | +55.86% |
|  | CD14 | 0.639 | 1.294 | +102.50% |
|  | CD163 | 0.74 | 0.907 | +22.57% |
|  | CD44 | 0.773 | 1.494 | +93.27% |
|  | MSR1 | 0.867 | 1.064 | +22.72% |
|  | STAT6 | 0.751 | 1.149 | +53.00% |
|  | ITGAL | 0.756 | 1.085 | +43.52% |
|  | GPNMB | 0.485 | 2.102 | +333.40% |
|  | CD300LF | 0.486 | 1.140 | +134.6% |
| **Category** | **Markers/Regulators** | **D3 VS D0** | **D14 VS D0** | **Change (D14 VS D3)** |
| Immune Checkpoints modulators | LGALS3 | 0.8 | 1.855 | +131.87% |
|  | LGALS3BP | 1.043 | 1.267 | +21.48% |
|  | TGFBI | 1.007 | 1.364 | +35.45% |
|  | TGFBR1 | 0.971 | 1.292 | +33.06% |
|  | CD38 | 0.424 | 1.174 | +176.89% |
|  | CD300LF | 0.486 | 1.140 | +134.6% |
|  | CD200 | 0.486 | 1.14 | +134.57% |
|  | GPNMB | 0.485 | 2.102 | +333.40% |

**Supplementary Table S6. Fold changes of markers/regulators for macrophages and immune checkpoints .**

| **Process** | **Gene**  **1** | **Gene**  **2** | **Gene**  **3** | **Gene**  **4** | **Gene**  **5** | **Gene**  **6** |
| --- | --- | --- | --- | --- | --- | --- |
| Apoptosis, Necrosis Necroinflammation, Pyroptosis, Ferroptosis | CASP2 | SERPINE1 | PTGS2 | ACOD1 |  |  |
| Metabolic reprogramming, ER Stress UPR | HK1 | PFKP | PKM | SLC2A1 | SLC2A3 | GPD2 |
| Fibrogenic remodeling, EMT Progenitor activation | MMP1 | MMP3 | VIM | FN1 | THBS3 | ANXA1 |
| Immune checkpoints tolerance | LGALS3 | TGFBI | CD300LF | CD200 | GPNMB |  |
| Dendritic cell activity, Phagocyte polarization (M1M2), Antigen presentation (MHC Ⅲ) | HLA-DRA | HLA-DRB1 | IGSF6 | CD86 |  |  |
| Developmental pathways, Neuroendocrine signaling, Exosome vesicle biology | SPHK1 | NOTCH1 | RAB27B | SDCBP | IGF1R |  |
| DNA repair Proliferation, Senescence | CDK2 | CDK12 | MCM10 | PCNA | TOP3A |  |
| Oxidative Stress | GSTA4 | GPX1 | SOD1 | EGLN1 | NRF1 |  |
| Complement/Coagulation, Acute-phase/Liver injury | C3 | C8A | C9 | F5 | F10 | SERPING1 |
| NKB cell activity, T cell activation | NCAM1 | GZMB | PRF1 | CD3E | CKLF |  |

**Supplementary Table S7. Canonical gene sets used for stouffer analysis.**
